# Evolving Approaches to Bacterial Identification: A Review of Classical and Modern Techniques

**DOI:** 10.3390/ijms27115092

**Published:** 2026-06-04

**Authors:** Ina Gajic, Milos Jovicevic, Dusan Kekic, Jovana Kabic, Ivan Vicic, Bojana Lukovic, Ana Tomic, Olja Sovljanski, Mila Skoric, Iva Sikanic, Marko Jankovic, Aleksandra Smitran, Ljiljana Bozic, Bojan Golic, Jasmina Basic, Nedjeljko Karabasil, Natasa Opavski

**Affiliations:** 1 Institute of Microbiology and Immunology, Faculty of Medicine, University of Belgrade, 11000 Belgrade, Serbia; milos.jovicevic@med.bg.ac.rs (M.J.); dusan_vk@yahoo.com (D.K.); jovana.kabic@gmail.com (J.K.); milaskoric192@gmail.com (M.S.); ivasikanic@gmail.com (I.S.); jankovic.marko1987@gmail.com (M.J.); natasaopavski@gmail.com (N.O.); 2 Department of Food Hygiene and Technology, Faculty of Veterinary Medicine, University of Belgrade, 11000 Belgrade, Serbia; ivan.vicic@vet.bg.ac.rs (I.V.); nedja@vet.bg.ac.rs (N.K.); 3 College of Health Sciences, Academy of Applied Studies Belgrade, 11000 Belgrade, Serbia; bojanall@yahoo.com (B.L.); basic.jasmina23@gmail.com (J.B.); 4 Faculty of Technology, University of Novi Sad, 21000 Novi Sad, Serbia; anav@uns.ac.rs (A.T.); oljasovljanski@gmail.com (O.S.); 5 Department of Microbiology and Immunology, Faculty of Medicine, University of Banja Luka, 78000 Banja Luka, Bosnia and Herzegovina; aleksandra.smitran@med.unibl.org (A.S.); ljiljana.bozic@med.unibl.org (L.B.); 6 Public Institution Veterinary Institute of the Republic of Srpska “Dr. Vaso Butozan”, 78000 Banja Luka, Bosnia and Herzegovina; bojan.golic@virs-vb.com

**Keywords:** bacterial identification, culture, diagnostic microbiology, polymerase chain reaction (PCR), whole-genome sequencing (WGS), metagenomics, MALDI-TOF MS, artificial intelligence in microbiology, antimicrobial resistance (AMR), rapid pathogen detection

## Abstract

Infectious diseases remain a major global health concern, with a growing burden of antimicrobial resistance and consequent higher mortality in the human population. Accurate bacterial identification is fundamental across clinical, veterinary, agricultural, and research settings, supporting effective diagnosis, antimicrobial stewardship, infection control, food safety, and environmental monitoring; however, conventional approaches are limited by time constraints, reduced sensitivity, and challenges in detecting fastidious or uncultivable organisms. This review provides a comprehensive overview of classical and advanced methods, including microscopy, culture, biochemical testing, immunological and serological assays, proteomic and spectroscopy-based techniques, and molecular approaches, such as polymerase chain reaction (PCR), digital PCR, DNA hybridization, 16S rRNA gene sequencing, whole-genome sequencing, and metagenomics. The integration of artificial intelligence has further enhanced analytical performance. Nevertheless, harmonization of bioinformatics frameworks remains essential, as variability in algorithm-defined cut-off values limits standardized implementation of whole-genome sequencing in routine laboratories. Emerging technologies, including CRISPR-based diagnostics and phage- and nanomaterial-based detection systems, offer promising alternatives. Overall, the integration of these approaches is expected to improve the accuracy, speed, and applicability of bacterial identification across diverse settings; however, these advances should be implemented cautiously, with standardization remaining a key priority alongside technological modernization.

## 1. Introduction

Infectious diseases remain a major global public health concern and represent one of the primary contributors to health loss worldwide, accounting for an estimated 13.7 million infection-related deaths (95% UI 10.9–17.1) in 2019 [1,2]. Of these, approximately 7.7 million deaths were linked to 33 bacterial pathogens [2]. Global analysis indicates that more than 39 million people around the world could die from antibiotic-resistant infections between 2024 and 2050 [3]. Furthermore, antimicrobial resistance (AMR) is widely projected to become a major contributor to mortality: the World Health Organization (WHO) estimates that 10 million deaths could occur yearly by 2050 [4].

Therefore, early and effective management of bacterial infectious diseases is a priority area that necessitates a multifaceted approach, including accurate microbiological diagnosis. The reliable identification of bacteria, encompassing genus, species, and occasionally strain-level resolution, is essential for early diagnosis, infection control, environmental monitoring, industrial quality assurance, food safety, pharmacy, and microbiological research. It ensures appropriate antimicrobial therapy, reduces the dissemination of infectious diseases, helps track the spread of pathogens, and supports quality control in industrial and healthcare settings.

Conventional bacterial identification, primarily relying on microscopy, culture-based techniques, and biochemical tests, still serves as a foundational practice worldwide. However, many bacteria share similar morphological and biochemical characteristics, which can lead to misidentification when relying solely on conventional methods. Some organisms are slow-growing, fastidious, or non-culturable under standard laboratory conditions, further complicating sensitive and accurate detection. Additionally, traditional biochemical tests can be time-consuming, leading to delayed results, and are subject to human error, particularly in laboratories with limited resources and time constraints. Further, unknown bacteria highlight the limitations of conventional identification, the need for molecular or proteomic approaches, and the importance of standardization to reduce misidentification. Although advanced molecular techniques such as polymerase chain reaction (PCR) and sequencing provide higher accuracy, their high cost, need for specialized equipment, and technical expertise limit their routine use in many educational and resource-limited laboratories. This creates a gap between the ideal methods for bacterial identification and the practical approaches commonly employed in routine microbiology laboratories. However, automated systems based on protein profiling and molecular identification have partially reduced the impact of the aforementioned limitations [5].

Despite its importance, bacterial identification remains challenging. In contrast to antimicrobial susceptibility testing (AST), which is highly standardized and harmonized through guidelines such as those of the Clinical and Laboratory Standards Institute (CLSI) and the European Committee on Antimicrobial Susceptibility Testing (EUCAST), procedures for identifying bacterial species are far less uniform. Of note, accurate species identification is essential for interpreting AST results, as breakpoints are species-specific. This raises an important question: can susceptibility testing truly be standardized if bacterial identification itself is not?

Given these challenges, there is a clear need to critically evaluate the laboratory techniques currently used for bacterial identification and to explore strategies for integrating novel, accurate, affordable, and practical methods into routine diagnostic workflows. Addressing these issues could enhance bacterial identification overall, enabling faster, more sensitive, and more reliable bacterial detection in relevant sectors. Accordingly, this review aims to examine the importance, current methodologies, limitations, and global practices of bacterial identification. Particular attention is given to methodological shortcomings that may affect diagnostic accuracy, clinical outcomes, and food safety.

## 2. Current Methods for Bacterial Identification

Methods for bacterial detection and identification are commonly classified as phenotypic or genotypic, and as culture-based or culture-independent. Classical methods, often referred to as traditional approaches, rely mainly on phenotypic, culture-based techniques, including microscopic examination, assessment of colony morphology, and biochemical tests evaluating metabolic and enzymatic activities.

### 2.1. Microscopic Examination of Bacteria

**Figure 1:**
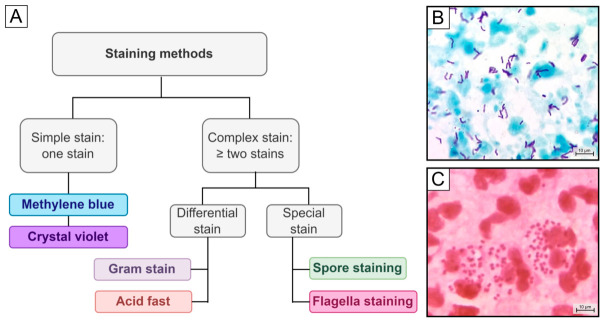
Staining methods used in bacteriology. (**A**): Classification; examples of differential stains: Gram stain; (**B**): Positive Ziehl-Neelsen stain, with acid-alcohol-resistant bacilli (ARB), Adapted from: [6]; (**C**): Intracellular Gram-negative *N. gonorrhoeae* diplococci, Adapted from: [7].

Although bacterial identification methods have advanced significantly, microscopic assessment remains important in microbiological diagnostics. A native smear, though rarely used in bacteriology, is an unstained preparation that enables rapid observation of microbial morphology, motility, and approximate quantity. In contrast, stained smears are colored and classified, with selected examples shown in Figure 1.

The Gram stain remains the most widely used staining technique and cornerstone of bacterial microscopy, distinguishing Gram-positive from Gram-negative bacteria based on cell wall composition, providing information on morphology (cocci, bacilli, and coccobacilli) and arrangements (chains, clusters, palisades, “Chinese letter,” or seagull-wing patterns) [8]. Gram staining of sterile-site smears, particularly in suspected sepsis or meningitis, rapidly guides early treatment decisions even ahead of molecular results [9].

Acid-fast (Ziehl–Neelsen) staining is a cost-effective primary tool for tuberculosis diagnosis and guides further analyses. Besides *Mycobacterium* species, other acid-alcohol-resistant bacilli (ARB) include *Nocardia* and *Rhodococcus*.

Special stains are highly specific techniques designed to highlight unique, specialized structures, such as capsules, flagella, or spores that are not present in all bacteria.

Microscopic examination of colonized sites (e.g., wound samples) helps distinguish true infection from colonization, as the presence of polymorphonuclear leukocytes indicates active infection. Gram-stained smears also differentiate true sputum from saliva: 10–25 squamous epithelial cells per low-power field indicate contamination, while leukocyte-rich, low-epithelial samples should be considered lower respiratory tract specimens and are suitable for culture [10]. However, sputum quality criteria vary, and a recent study suggests that culturing all samples, regardless of microscopic quality, may still yield valuable diagnostic information in community-acquired pneumonia [11].

Microscopy alone can provide a final diagnosis in selected cases: in bacterial vaginosis, vaginal Gram stain, the reference method, quantifies lactobacilli, small Gram-negative and Gram-variable rods (i.e., *Gardnerela vaginalis* or *Bacteroides*), and curved Gram-negative rods (i.e., *Mobiluncus*): Nugent scores of 0–3 indicate *Lactobacillus* predominance, 4–6 intermediate flora, and 7–10 bacterial vaginosis [12]; in men, urethral discharge microscopy can confirm symptomatic gonorrhea, as Gram-stained smears showing polymorphonuclear leukocytes with intracellular Gram-negative diplococci are highly specific and sensitive and can be considered diagnostic for *Neisseria gonorrhoeae* infection in symptomatic cases [13].

However, detection thresholds in microscopy vary: Ziehl–Neelsen staining for *M. tuberculosis* requires ~10^4^ bacilli/mL, Gram staining 10^5^–10^6^ bacteria/mL, and urethral smears for gonorrhea > 10–10^2^ intracellular diplococci per oil-immersion field. While rapid and inexpensive, microscopy is less sensitive than culture or molecular methods and is operator-dependent, though automation improves reproducibility and turnaround time [14].

### 2.2. Bacterial Cultures

**Figure 2:**
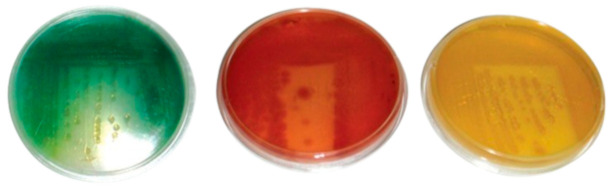
Pure bacterial cultures of *Pseudomonas* spp. (different pigments production on Mueller–Hinton agar, 37 °C, aerobic conditions, 24 h incubation). Adapted from: [15].

Clinical microbiology laboratories use culture-based methods to grow bacteria, enabling isolation and preliminary identification based on metabolic and nutritional characteristics. Culture media may be liquid (broth), solid, or biphasic (e.g., blood culture bottles) and are generally classified as basal, enriched, selective, differential, or chromogenic, with some media combining multiple properties. Commonly used solid bacterial culture media and broths, along with their properties, are presented in Supplementary Table S1 and Supplementary Table S2, respectively. Colony features, morphology, color, and sometimes odor aid identification. For instance, *Pseudomonas* spp. produce pigments (Figure 2), *Serratia marcescens* develops red colonies at room temperature, *Proteus* spp. show swarming growth with a putrid odor, and *Bacillus anthracis* forms non-hemolytic “medusa-head” colonies.

Bacterial growth depends on O_2_, CO_2_ levels, and temperature; typical requirements and incubation times are summarized in Table 1.

While culture-based methods remain central to bacterial identification, primarily because they enable AST on isolated strains, certain organisms cannot be cultivated using bacterial culture media, including *Mycobacterium leprae*, *Treponema pallidum*, and obligate intracellular bacteria like *Chlamydia* spp. and *Rickettsia* spp. Some pathogens are slow-growing, such as *Mycobacterium tuberculosis* and *Legionella pneumophila*. Such limitations underscore the importance of complementary approaches, including microscopy, antigen detection, and molecular assays, to ensure timely and accurate diagnosis.

### 2.3. Biochemical Tests

Bacteria produce characteristic sets of enzymes and metabolites that biochemical tests exploit for identification and preliminary differentiation. These assays detect enzyme–substrate reactions, often via color change, using minimal culture and yielding results within minutes to 24 h. A wide range of tests is in use (Table 2), with catalase, oxidase, coagulase, and urease among the most commonly employed [17].

The catalase test detects the enzyme catalase and is commonly used to differentiate catalase-positive *Micrococcaceae* from catalase-negative *Streptococcaceae*. It also aids species identification (e.g., *Aerococcus urinae*-positive vs. *Aerococcus viridans*-negative), certain catalase-positive *Campylobacter* species, and helps distinguish aerobic bacteria from obligate anaerobes (e.g., *Bacillus*-positive vs. *Clostridium*-negative) [18,19]. Most mycobacterial species are catalase-positive; however, *Mycobacterium tuberculosis* and *M. bovis* are typically negative in the 68 °C heat-stable catalase test, which may aid in their differentiation from other mycobacteria. Among slow-growing nontuberculous mycobacteria (NTM), heat-stable catalase is not produced by *M. marinum*, *M. avium* complex, *M. haemophilum*, *M. gastri,* and *M. shimoidei.* The semiquantitative catalase test may also contribute to the identification of certain slow-growing and rapid-growing NTM [16]. The catalase test is also useful in the preliminary differentiation of catalase-positive members of the *Enterobacteriaceae* family and *Listeria* spp. from morphologically similar catalase-negative streptococci and enterococci [16]. False-negative results may occur with old cultures, contaminated samples, or blood agar interference, while false-positive results may arise from technique-related artifacts, such as vigorous mixing or residual bubbles.

Free coagulase, beyond differentiating *Staphylococcus aureus* from coagulase-negative staphylococci, plays a key role in virulence by activating host prothrombin to form fibrin clots that protect bacteria from immune defenses and support biofilm formation.

The oxidase test detects cytochrome oxidase, primarily used to distinguish oxidase-positive *Pseudomonaceae* from oxidase-negative *Enterobacteriaceae*, among other applications. Spontaneous reagent oxidation or the use of metal loops can cause false positives, so fresh colonies and non-metallic loops are recommended.

The urease test (broth: Rustigian–Stuart; agar: Christensen) detects urease activity, enabling rapid identification of urease-positive organisms, including *Helicobacter pylori* in gastric biopsies, and supporting differentiation of certain Gram-negative coccobacilli. Slow growth or low enzyme expression may cause weak or delayed color change, while over-incubation can yield nonspecific results; accurate interpretation requires fresh reagents, proper colony selection, and controlled incubation.

Some bacteria (e.g., *E. coli*) produce indole from tryptophan via tryptophanase [20].

**Figure 3:**
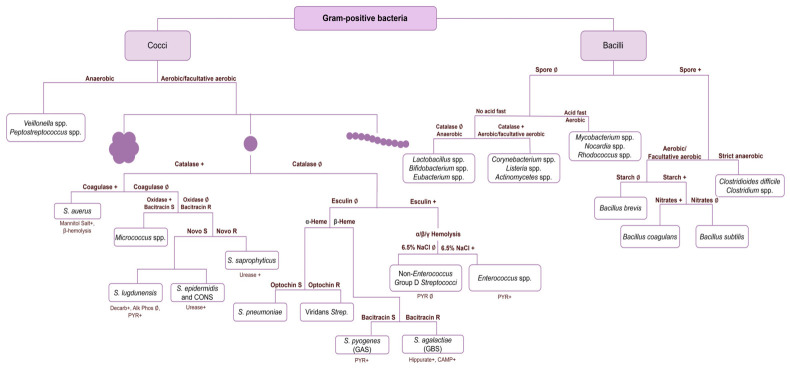
Identification algorithm for Gram-positive bacteria; +: positive; Ø: negative.

**Figure 4:**
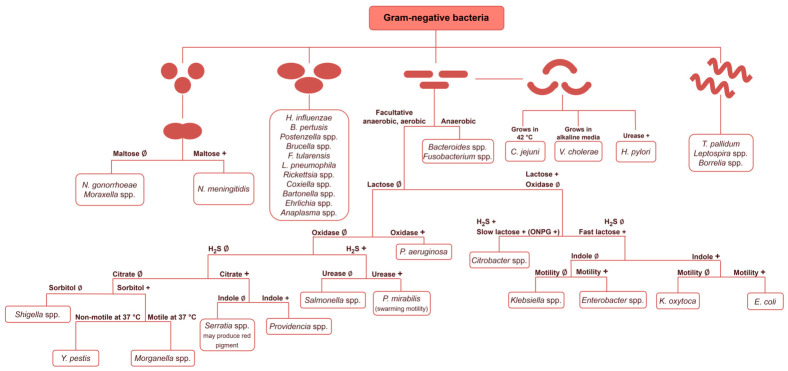
Identification algorithm for Gram-negative bacteria; +: positive; Ø: negative.

A common commercial identification system is the Analytical Profile Index (API, bioMérieux, Marcy-l’Étoile, France), which miniaturizes biochemical tests into scored reactions interpreted by a “best fit” scoring system to generate an analytical profile. The BD BBL™ Crystal™ identification system (Becton Dickinson, Sparks, MD, USA) is a similar variation of the API system [21]. Major biochemical features of Gram-positive and Gram-negative bacteria are summarized in identification algorithms (Figure 3 and Figure 4).

## 3. Immunological Methods, Including Serology, in Bacterial Detection

Immunological methods detect bacteria via antigen–antibody reactions. Serology, in the narrow sense, refers specifically to antibody detection. Together, they enable rapid and specific identification of bacteria and their differentiation at the species or strain level.

### 3.1. Agglutination Tests

Agglutination methods identify conserved bacterial antigens or antibodies and can be divided into direct, such as slide agglutination, and indirect or passive, including latex agglutination, biosensor-based assays, and passive haemagglutination. Slide agglutination identifies bacteria by directly mixing colonies with specific antisera on a slide. Latex agglutination detects bacterial antigens through visible clumping between antibody-coated beads and bacterial cells. Coagglutination employs antibodies bound to carrier particles, such as protein A-coated cells, to detect bacterial antigens, and passive haemagglutination, such as TPHA, detects antibodies against pathogens like *T. pallidum*. Agglutination tests require fresh reagents and optimal colonies. Results may be compromised by low antigen levels, degraded antibodies, or improper colony selection. Targets, advantages, and limitations of these methods are summarized in Table 3.

### 3.2. Enzyme-Linked Immunosorbent Assay

Enzyme-linked immunosorbent assay (ELISA) is widely used to detect antibodies in sera (indirect or competitive ELISA) or to identify bacteria directly (direct and sandwich ELISA). In direct ELISA, immobilized antibodies bind the target antigen, and a chromogenic substrate produces a color change for detection. This method allows rapid pathogen detection in cerebrospinal fluid and is used in commercial kits, such as Rapid Colorimetric Kits (Rosco Diagnostica A/S, Taastrup, Denmark), for pathogen identification and antimicrobial resistance testing [22].

“Sandwich” ELISA is a highly sensitive and specific method for detecting bacteria in liquid samples, using a capture antibody immobilized on a microtiter plate and a secondary enzyme-labeled antibody to form an antibody–antigen “sandwich,” with signal intensity proportional to bacterial concentration. Its main limitation is its relatively high detection threshold, often requiring high bacterial loads (e.g., 10^5^–10^7^ CFU/mL for *E. coli* O157:H7) [23]. Improved multi-antibody formats lower the detection limit to about 10^2^–10^3^ CFU/mL, enabling detection of low bacterial concentrations in food and environmental samples, including *Listeria monocytogenes* [24].

ELISA is often preferred over traditional techniques due to its rapid turnaround time and suitability for high-throughput automation, but manual performance requires expertise and may affect reproducibility. An additional advantage of ELISA is that it provides quantitative results, enabling direct comparison between samples. Limitations include: dependence on antibody quality and storage; nonspecific binding may yield a higher rate of false-positive results compared to other techniques; low bacterial load leading to false negatives, often requiring pre-enrichment and prolonged cultivation to reach the detection threshold; variable antigen expression; and the cost and complexity of reagent production, which limit availability and routine use to common pathogens [25].

### 3.3. Western Blot

Western blot (immunoblotting) is a confirmatory serologic technique detecting pathogen-specific antibodies or, less commonly, antigens after SDS–PAGE separation and membrane transfer. In *Borrelia burgdorferi* infection, it confirms positive or equivocal screening results by assessing reactivity to defined bands (e.g., OspC, p41, VlsE), with IgM indicating early and IgG multiple-band reactivity supporting later disease [26].

Weak signals may result from low protein load, inefficient transfer, or poor antibody binding; high background from inadequate blocking or excess antibody; nonspecific bands from cross-reactivity; and poor resolution from gel or loading issues.

### 3.4. Flow Cytometry

Flow cytometry (FCM) detects fluorescence from labeled probes to analyze cellular properties and identify specific populations. In microbiology, it enables rapid characterization of microorganisms, assessment of viability, pathogen detection, and monitoring of immune responses, thereby improving infection management [27].

Alongside Gram staining and culture, FCM complements routine diagnostics by providing rapid bacterial quantification [28]. It has also been applied to detect and differentiate carbapenemase types in enterobacteria [29]. Because bacteria may enter a viable but nonculturable state, culture can underestimate true counts, whereas FCM with viability probes enables sensitive detection of metabolically active cells [30]. For high-throughput single-cell detection and characterization of microorganisms, FCM can be combined with fluorescence in situ hybridization (Flow-FISH), which uses highly specific DNA or (m)RNA probes targeting the microorganism or transcript of interest [31].

Although FCM shows promise for rapid diagnostics, including antimicrobial resistance detection and the screening of clinical samples prior to further identification methods, its routine use in microbiology is limited by the lack of specific bacterial antibodies, difficulty distinguishing microbes from cellular debris, and sample aggregation. High cost, complexity, and the need for specialized expertise further restrict its use mainly to research and specialized or high-throughput laboratories rather than routine diagnostics [27,32].

### 3.5. Urinary Antigen Detection in Pathogen Identification

Immunological assays based on urinary antigen testing (UAT) are widely used for rapid detection of soluble bacterial antigens excreted in urine. The method is based on antigen–antibody interactions, most commonly using immunochromatographic membrane assays, although fluorescence immunoassays are also available. Urine passes along a membrane containing antibodies against target bacterial antigens, and antigen binding generates a visible signal.

Commercial assays are primarily available for detection of *Streptococcus pneumoniae* and *Legionella pneumophila* antigens in patients with community-acquired pneumonia (CAP) [33]. UAT tests are designed to detect antigens common to all serotypes of *S. pneumoniae* and antigens of *L. pneumophila* serogroup 1, which account for approximately 80–95% of legionellosis cases. The most widely used method is the immunochromatographic membrane assay (e.g., BinaxNOW, Abbott, IL, USA) [33]. To improve the diagnostic coverage of *Legionella* UAT, novel assays such as the Ribotest (Asahi Kasei Pharma, Tokyo, Japan) have been developed, enabling detection of all *L. pneumophila* serogroups [34]. Furthermore, high-throughput multiplex urinary antigen assays using serotype-specific monoclonal antibodies and Luminex microfluidics platforms have been developed for rapid simultaneous detection of pneumococcal serotypes included in PCV15 [35].

Compared with conventional microbiological methods, which often fail to identify the causative pathogen and therefore necessitate empiric therapy, UAT offers several advantages, including rapid turnaround time, non-invasiveness, high specificity (>95%), and moderate sensitivity (approximately 75–85%) [33]. These characteristics enable timely pathogen-directed therapy and may reduce complications associated with empiric antimicrobial treatment, including antimicrobial resistance and *Clostridioides difficile* infection. Accordingly, the European CAP guidelines recommend UAT use in appropriate clinical settings [36]. However, important limitations remain. Diagnostic performance is generally better in moderate-to-severe CAP than in mild disease, and pneumococcal UAT may yield false-positive results in children; therefore, its routine use in pediatric populations is generally not recommended.

### 3.6. Lateral Flow Immunoassay

Lateral flow immunoassay (LFIA) is a chromatography-based technique that uses capillary flow of a liquid sample through device components, including the sample pad, conjugate pad, nitrocellulose membrane, absorbent pad, and backing layer. This platform is a rapid (5–20 min), point-of-care tool for on-site pathogen detection. The assay relies on antibodies or alternative bioreceptors targeting specific microbial epitopes. Antigen–bioreceptor binding is visualized as a test line, while a control line confirms proper sample migration and conjugate release [37].

Commercial LFIA-based devices (e.g., BD Veritor™ Plus, Becton Dickinson and Company, Sparks, MD, USA; Sofia^®^ Strep A+ Fluorescent Immunoassay, QuidelOrtho Corporation, San Diego, CA, USA; OSOM^®^ Strep A, Sekisui Diagnostics, San Diego, CA, USA) can detect *S. pyogenes* from throat swabs with ~70–95% sensitivity and >90% specificity, depending on sample quality and bacterial load. Multiplex formats improve specificity by targeting multiple strains simultaneously. Similar platforms are available for non-invasive detection of *H. pylori* stool antigens, often exceeding 90% sensitivity and specificity (e.g., STANDARD™ F *H. pylori* Ag FIA, SD Biosensor, Suwon, Gyeonggi-do, Republic of Korea) [38].

Recent advancements include nanozyme-based labeling, improving signal amplification, stability, diagnostic accuracy, and usability [38]. Thus, LFIA systems are increasingly incorporating nanoparticles instead of conventional antibodies. For instance, silver nanoparticle-enhanced LFIA enables detection of *Salmonella enterica* and *E. coli* O157:H7 in meat samples at levels as low as 1–10 CFU/g [39]. Aptamers, synthetic DNA/RNA “chemical antibodies”, are emerging as stable, cost-effective alternatives to conventional antibodies [39].

Ongoing developments focus on improving sensitivity, specificity, and multiplexing, as well as integrating digital and mobile technologies for real-time analysis and remote diagnostics, expanding LFIA applications in healthcare, environmental monitoring, and food safety [37].

## 4. Proteomic and Spectroscopy-Based Methods

### 4.1. MALDI-TOF Mass Spectrometry

Matrix-Assisted Laser Desorption Ionization–Time of Flight Mass Spectrometry (MALDI-TOF MS) is a rapid automated method for bacterial identification based on mass analysis of ionized bacterial proteins, mainly conserved ribosomal proteins. After co-crystallization with an organic matrix and laser ionization, characteristic mass spectra (“fingerprints”) are generated and compared with reference databases for species identification. MALDI-TOF MS has revolutionized clinical bacteriology by enabling identification from cultured isolates or positive blood cultures within minutes, compared with the 24–48 h required for conventional biochemical methods. This is a great advantage, especially in the identification of anaerobes, fastidious microorganisms like Gram-negative bacteria of the HACEK group, and slow-growing pathogens like *Mycobacterium* spp., *Leptospira* spp., or *Borrelia* spp. Using different approaches, MALDI-TOF MS can also detect antimicrobial resistance biomarkers, including beta-lactamase, carbapenemase, and cephalosporinase activity. The method offers high sensitivity, throughput, simple operation, and low cost per sample, although instrumentation and maintenance are expensive [40]. The analytical sensitivity of MALDI-TOF MS may vary when applied directly to biological samples. Direct analysis from blood culture bottles requires sample preparation using either commercial kits or in-house centrifugation and lysis protocols prior to analysis, often in combination with the Sepsityper software module (MALDI BioTyper Library ver. 11.0.0.0) [41]. Additionally, different studies have reported the successful bacterial identification directly in cerebrospinal fluid and urine samples [42,43].

Nevertheless, MALDI-TOF MS may show limited discriminatory power for closely related species (e.g., *S. pneumoniae* vs. *S. mitis*/*oralis*, *E. coli* vs. *Shigella* spp., or members of the *M. tuberculosis* complex). Identification may also be influenced by growth conditions and sample preparation, particularly in spore-forming or heavily encapsulated organisms such as *Klebsiella pneumoniae*, although formic acid extraction can improve performance. Overall accuracy largely depends on the quality and comprehensiveness of spectral databases, which continue to evolve through novel spectral approaches that improve differentiation, including between *S. parapneumoniae* and *S. pneumoniae* [44], as well as through further integration with molecular reference data. The method requires sufficient biomass from pure culture and is unreliable for mixed samples [40].

### 4.2. FTIR and Raman Spectroscopy

Fourier transform–infrared (FT-IR) and Raman spectroscopies are widely used across technological and industrial applications for the chemical identification of materials. The result of their work is today’s modern systems for microbial characterization, like IR Biotyper (Bruker GmbH, Bremen, Germany), which enables a broad spectrum of uses, from final identification, classification, and typing to analysis of transmission routes and infection control [45]. The addition of different data processing programs, artificial intelligence, and machine learning leads to a broad-spectrum expansion of applications of this technology.

The principles of the methods are based on the sample’s irradiation by infrared light, which interacts with molecules, causing absorption of the spectrum (FT-IR) or scattering (Raman) that is unique to the molecule. The spectrum is recorded and analyzed, providing biochemical fingerprinting of the chemical composition of the sample (including carbohydrates, proteins, lipids, DNA, and RNA). It is a highly sensitive and specific method that enables nearly similar typing information as molecular methods, depending on the capabilities of the current database [46]. The IR and Raman spectroscopies are complementary, since dipole-moment-sensitive functional groups better react to IR, while functional groups with low sensitivity to dipole moments are more responsive to the Raman method [47].

In modern microbiology, IR Biotyper and related techniques can be applied in science-based studies to explore the composition of microbial cells and biofilms, as well as the effects of various compounds on their structures [48]. They also have broad applications, including veterinary use and food production management and safety. For example, *Salmonella* typing and tracking, *E. coli* subtyping in meat production, and *L. monocytogenes* serogroup analysis in food samples; probiotic production and classification; hygiene monitoring in factories and hospitals; and clinical applications, such as tracking antibacterial resistance, clonal types of emerging bacteria, vaccine-relevant pneumococcal serotypes, accurate differentiation and identification of specific bacteria (e.g., *E. coli*, *Shigella*, and *Mycobacterium* spp.), differentiation of commensal and pathogenic oral bacteria, and outbreak investigations in clinical intensive care units or food-borne outbreaks [45,48,49,50,51,52,53,54,55].

The use of this technology is easy, as it does not require much sample preparation, and is faster and far less expensive than molecular techniques. It is proposed as a good real-time surveillance system for public health, clinical settings, or food safety (one health system) as a screening method, followed by more complex and accurate molecular analysis, such as whole-genome sequencing (WGS) [46].

Currently, this technology is still not widely available or routinely used, and its performance remains highly dependent on the quality and comprehensiveness of reference databases. In addition, as with many emerging technologies, it still has certain limitations in fine-scale bacterial characterization, for example in distinguishing closely related pneumococcal serotypes, such as 6A, 6B, and 6C [45,50].

## 5. Molecular Methods for Bacterial Identification

The introduction of molecular technologies has markedly transformed microbiological diagnostics, enabling rapid and accurate detection of pathogens and antimicrobial resistance, particularly in non-cultivable, fastidious, or environmentally sensitive organisms. Major molecular techniques used are displayed in Supplementary Table S3.

### 5.1. PCR-Based Methods for Bacterial Identification

PCR-based assays are widely used in clinical, research, and other microbiological laboratories due to their high sensitivity, specificity, and rapid turnaround time, enabling direct detection of bacterial nucleic acids and characterization of virulence and resistance determinants.

Conventional PCR is primarily used for single-target, species-specific gene detection. Multiplex PCR extends diagnostic capacity by enabling simultaneous amplification of multiple targets within a single reaction, thereby improving diagnostic efficiency in syndromic testing. Advanced primer and probe designs reduce primer–dimer formation and competitive amplification. Recent applications include multiplex panels for lower respiratory tract and abdominal pathogens, and portable devices capable of detecting multiple oral pathogens simultaneously [56,57]. Emerging multiplex implementations, such as PCR–dipstick DNA chromatography, demonstrate the potential for rapid (~40-min) and specific detection of multiple respiratory bacteria in clinical samples [58].

Real-time or quantitative PCR (qPCR) enables real-time amplification and quantification of bacterial DNA through fluorescent signal monitoring (e.g., SYBR Green or TaqMan) in a closed system, enabling automation. Multiplex qPCR allows simultaneous detection of multiple targets using distinct fluorophores and is widely used in respiratory and sepsis diagnostics [59,60].

Lab-on-chip PCR integrates PCR into microfluidic platforms, enabling miniaturized diagnostics, particularly suitable for mixed infections [61], in point-of-care settings.

#### 5.1.1. Digital PCR Technology

Limitations of first-generation classical end-point PCR and second-generation qualitative PCR and qPCR, particularly in sensitivity, are overcome by third-generation technologies, such as digital PCR (dPCR) and droplet digital PCR (ddPCR) [62]. dPCR partitions samples into numerous single-molecule nucleic acid compartments and uses fluorescent probes or dyes for amplification, enabling accurate absolute quantification without standard curves compared to qPCR [63]. ddPCR further enhances sensitivity by dividing the reaction mixture into thousands of droplets, followed by end-point amplification and fluorescence-based droplet analysis using FCM [64]. This technology offers clinical benefits, including early diagnosis of invasive infections and detection of resistance mechanisms, as well as public health applications, such as wastewater and waterborne pathogen monitoring [63]. However, its routine implementation remains limited by more complex data analysis; qPCR remains the gold standard for detection and quantification due to its simplicity and reproducibility [64]. Another variation of ddPCR, ddPCR coupled with melting curve analysis (ddPCR-MCA), integrates DNA amplification, quantification, and sequence discrimination in a single workflow [65]. Following amplification, PCR products are subjected to melting curve analysis, where the melting temperature reflects sequence composition and enables differentiation of closely related bacterial species, detection of sequence variants, and identification of nonspecific products. ddPCR-MCA enhances sensitivity and provides absolute quantification of low-abundance bacterial targets, improving detection in complex or inhibitor-rich samples, though accuracy remains around 85% and requires further optimization.

#### 5.1.2. Loop-Mediated Isothermal Amplification and CRISPR-Based Molecular Diagnostics

Loop-mediated isothermal amplification (LAMP) amplifies nucleic acids at a constant temperature, unlike PCR, which requires thermal cycling. LAMP is an easy-to-read (often via color change), sensitive, and specific alternative for bacterial identification. However, because it relies on multiple primer sets, multiplexing is challenging, and assays targeting fewer sequences generally achieve higher specificity and sensitivity [66]. LAMP assays have been extensively used to identify clinically significant bacteria, including *Acinetobacter baumannii*, *K. pneumoniae*, *M. tuberculosis*, *S. aureus*, and *Pseudomonas aeruginosa* [67]. In addition to LAMP, several newer isothermal amplification techniques have emerged. Cross-priming amplification (CPA) is an isothermal technique that employs specially designed cross-primers and strand-displacement amplification to quickly amplify target DNA without needing thermal cycling. Known for its simplicity, rapid results, and minimal equipment requirements, CPA is ideal for field diagnostics and settings with limited resources [68,69]. Recombinase polymerase amplification (RPA) and recombinase-aided amplification (RAA) utilize recombinase enzymes, single-stranded DNA-binding proteins, and strand-displacing polymerases to amplify DNA at relatively low temperatures (37–42 °C). RPA/RAA reactions are rapid, often producing detectable results within 30 min [70]. They can also be integrated with lateral flow assays, fluorescence detection, or clustered regularly interspaced short palindromic repeats (CRISPR)-based platforms for sensitive pathogen detection.

The CRISPR/CRISPR-associated proteins (Cas) system is an adaptive immune mechanism found exclusively in bacteria and archaea, enabling them to defend against the intrusion of foreign nucleic acids. Owing to its streamlined design, exceptional editing efficiency, and high specificity toward target gene sequences, the CRISPR/Cas system holds significant promise as a powerful tool for bacterial detection [71]. The integration of the CRISPR/Cas system with diverse nucleic acid amplification techniques, such as loop-mediated isothermal amplification (LAMP), PCR, recombinase-aided amplification (RAA), recombinase polymerase amplification (RPA), and rolling circle amplification (RCA), and various signal output models, including colorimetric, fluorescence-based, Chemiluminescence Resonance Energy Transfer (CRET), surface-enhanced Raman scattering (SERS), and electrochemical platforms, has been described as a new-generation approach for detecting pathogenic bacteria that has significantly enhanced the sensitivity and efficiency of pathogenic bacterial detection [72,73,74,75]. Recent advances have further expanded the CRISPR-based diagnostic toolbox, particularly through the development of CRISPR/Cas12a systems, which offer strong collateral cleavage activity and improved signal amplification performance, thereby increasing diagnostic sensitivity and versatility. In addition, several CRISPR-based diagnostic platforms, such as SHERLOCK (https://www.broadinstitute.org/news/sherlock-team-advances-its-crispr-based-diagnostic-tool (Accessed on 7 February 2026)) and DETECTR (https://mammoth.bio/diagnostics/?utm_source=chatgpt.com (Accessed on 7 February 2026)), have been evaluated in clinically relevant settings or early translational studies for the detection of important bacterial pathogens, including *M. tuberculosis* and carbapenem-resistant Enterobacteriaceae, demonstrating rapid and highly sensitive detection with strong potential for future clinical implementation.

### 5.2. DNA Hybridization

DNA hybridization is one of the earliest molecular approaches used for bacterial identification and taxonomic classification, based on nucleic acid complementarity, where labeled probes bind target sequences. DNA–DNA hybridization was historically the reference standard for species delineation (≥70% relatedness) [76]. Current formats include direct probe assays, reverse hybridization line-probe assays, microarray platforms, bead-based suspension arrays, and probe-based real-time PCR detection systems.

In clinical diagnostics, hybridization principles have been translated into practical and standardized platforms. Early rRNA-targeted probe assays, such as the AccuProbe^®^ system (Hologic Inc., San Diego, CA, USA), enabled species identification directly from culture isolates, particularly for *Mycobacterium* spp. [77]. Reverse hybridization line-probe assays, including the GenoType^®^ series (Hain Lifescience GmbH, Nehren, Germany) and INNO-LiPA^®^ (Fujirebio Europe N.V., Ghent, Belgium), further expanded clinical applications by enabling species differentiation and detection of resistance-associated mutations [78]. Similar membrane-based systems, such as HybriSpot^®^ (Vitro S.A., Seville, Spain), have been applied for the targeted detection of species-specific genes for identification and antimicrobial resistance genes. Platforms such as the Verigene^®^ System (Luminex Corporation, Northbrook, IL, USA) and Luminex xTAG^®^/NxTAG^®^ (Luminex Corporation, Austin, TX, USA) assays employ bead-based or chip-based multiplex hybridization strategies to identify bacterial pathogens and selected antimicrobial resistance determinants directly from positive blood cultures and other clinical specimens [79,80].

Hybridization methods are inherently target-dependent, requiring predefined probes and prior knowledge of targets. Recently, they have been integrated into next-generation sequencing workflows for targeted enrichment of pathogen and resistance genes prior to sequencing [76].

### 5.3. Ribotyping

Ribotyping is a molecular typing method based on RFLP analysis of rRNA operons. Genomic DNA is digested, and fragments are hybridized with rRNA probes targeting conserved regions to generate strain-specific fingerprints. Automated systems, such as the RiboPrinter^®^ Microbial Characterization System (DuPont Qualicon, Wilmington, DE, USA), have enabled standardized inter-laboratory comparisons and have been extensively applied in surveillance and outbreak investigations.

For *C. difficile*, PCR ribotyping targets the 16S–23S rDNA intergenic spacer, with fragment patterns defining ribotypes (RTs). Although less discriminatory than genome-based methods, ribotyping remains widely used in Europe and North America for epidemiology, virulence, and resistance monitoring, and tracking the global prevalence of circulating strains [81].

### 5.4. Sequencing Methods

Sanger sequencing is used for bacterial identification by sequencing target genes (e.g., 16S rRNA or housekeeping genes), enabling species-level identification.

#### 5.4.1. 16S rRNA Gene Sequencing

The 16S ribosomal RNA (rRNA) gene is a universal bacterial target with conserved and hypervariable regions, enabling broad detection, phylogenetic classification, and taxonomic discrimination [56,70]. Sequencing of variable regions (V1–V9) supports high-throughput analyses and has been fundamental to the development of the three-domain system of life [69,71]. In clinical microbiology, 16S rRNA gene sequencing is primarily used for bacterial identification when conventional phenotypic or automated methods yield inconclusive results, particularly in culture-negative infections, such as infective endocarditis. In suspected cases, blood cultures are positive in ~55%, whereas 16S rRNA PCR/sequencing of valve tissue reaches ~75% positivity. After antibiotic treatment, culture sensitivity drops to ~11%, while molecular detection remains high (~76%) [72]. This approach also enables species-level identification and detection of previously unrecognized pathogens (e.g., *Eggerthella* spp.) [73].

Nevertheless, its limited discriminatory power among closely related species necessitates the use of additional housekeeping genes in multilocus approaches to improve resolution [82]. Beyond diagnostics, 16S rRNA sequencing is a cornerstone of microbiome research, with long-read platforms now enabling full-length sequencing and improved species-level resolution [83].

#### 5.4.2. Whole-Genome Sequencing (WGS)

Whole-genome sequencing (WGS), enabled by advances in second- and third-generation sequencing, allows high-resolution differentiation of closely related bacterial strains and the comprehensive identification of virulence factors and antimicrobial resistance determinants [84,85]. Genomic relatedness can be assessed using single-nucleotide polymorphism (SNP) analysis based on reference alignment and mutation detection [86]. Although SNP analysis provides high-resolution insight into genome structure, particularly in clonal species, multilocus sequence typing (MLST) offers a gene-by-gene alternative that identifies genetic variation across the core genome (cgMLST) or expanded gene sets, such as whole-genome MLST (wgMLST) [87].

WGS, as a culture-dependent technique, in practical terms, allows for a deeper understanding of the emergence and spread of pathogenic microorganisms, which, together with phylogenetic analysis, enables the discovery of sources and routes of pathogen transmission, especially in food-borne outbreaks, facilitating risk assessment, i.e., hazard and risk characterization [88].

A key limitation of WGS is that detected genes may not be phenotypically expressed and depend on reference databases [89]. In addition, the lack of standardized bioinformatics tools and cut-off criteria limits routine laboratory implementation [89,90].

#### 5.4.3. Metagenomics: Culture-Independent Analysis of Microbial Communities

Metagenomic analysis, a culture-independent technique, involves isolating total DNA from a sample without culturing specific microorganisms and examining microbiome diversity across matrices such as soil, food, and water [91]. A simpler metagenomic analysis, amplicon sequencing, based on the hypervariable 16S rRNA region in bacteria, allows taxonomic profiling to the genus level and the potential to predict the metabolic pathways of the identified bacteria [92]. A more complex approach, shotgun metagenomics, allows the sequencing of entire genomes, the identification of bacteria at the species level, and the determination of diverse genes for survival, host interaction, virulence factors, antimicrobial resistance, and the presence of mobile genetic elements for the identified bacterial species in a single DNA sample [93].

Shotgun metagenomics, although in practical terms it gives us insight into the distribution of important genes, primarily resistance genes, in bacterial species, both pathogenic and non-pathogenic, across different matrices, has limited applicability due to high costs, difficult interpretation, and the need for bioinformatics knowledge [94,95]. However, with the continuous decrease in sequencing costs and the increasing availability of automated analysis pipelines (e.g., QIAGEN CLC Genomics Workbench (QIAGEN, Hilden, Germany) and Illumina DRAGEN Bio-IT Platform (Illumina Inc., San Diego, CA, USA)), its application in clinical diagnostics is steadily expanding. Furthermore, when detecting pathogenic microorganisms and viable but non-culturable bacteria, sensitivity and specificity can be variable due to their possible low abundance in the sample; the main drawback is the potential to detect DNA from dead microorganisms [95,96]. Additionally, the metagenomic approach is increasingly applied in clinical microbiology through metagenomic next-generation sequencing (mNGS) and targeted next-generation sequencing (tNGS), with the development of automated analysis pipelines, especially in severe infections of the central nervous system and lungs and in sepsis. mNGS allows the identification of all bacteria, viruses, parasites, and fungi and their genetic characterization in clinical samples. On the other hand, a cost-effective alternative, tNGS, enables the identification of specific microorganisms of interest by prior PCR amplification of low-level pathogen spectra, facilitating rapid diagnostics in clinical settings [97].

#### 5.4.4. Long-Read Sequencing: Application in Bacteriology

Third-generation sequencing (TGS), known as single-molecule long-read sequencing, provides reads over 1000 bp, most commonly from 10 kbp to over 100 kbp, overcoming the limitations of next-generation sequencing (NGS), with reading fragments up to 600 bp, and more complex de novo genome assembly following prior PCR amplification [95,98]. TGS involves real-time monitoring of base incorporation during sequencing, with the potential to detect structural changes and epigenetic base modifications after bioinformatic processing. Portable long-read sequencing devices (e.g., Oxford Nanopore sequencing (Oxford Nanopore Technologies plc, Oxford, UK)) further allow real-time WGS, advancing rapid public health response [84,95]. High sequencing error rate (5–20%), in contrast to second-generation (<1%), and the large amount of post-processed data are the main disadvantages of TGS [95,99]. The integration of NGS and TGS through WGS and a metagenomic approach could overcome their limitations and more precisely identify and characterize hazards during epidemiological outbreaks, as well as improve disease diagnostics in clinical settings.

## 6. Automation and Integrated Systems

Automated and integrated systems may be based on biochemical profiling, protein analysis (see Section 4.1), or molecular approaches.

### 6.1. Automated Biochemical-Based Platforms

Automated diagnostic platforms, like VITEK 2 (bioMérieux, Marcy-l’Étoile, France), BD Phoenix (Becton, Dickinson and Company, Franklin Lakes, NJ, USA) and MicroScan WalkAway Systems (Beckman Coulter, Inc., Brea, CA, USA), streamline bacterial identification and AST by reducing manual work and providing standardized results. All require a pure colony as input. The instruments incubate and monitor growth, and the software interprets the data to provide identification, AST, and minimum inhibitory concentrations (MICs). All three systems use automated broth microdilution for AST. VITEK 2 detects growth via turbidity (optical density changes); BD Phoenix uses redox indicator color change or turbidity; and MicroScan WalkAway measures photometric changes in turbidity. VITEK 2 and BD Phoenix can generate results within the same working day, while MicroScan WalkAway often requires overnight incubation [100,101]. Limitations include dependence on pure cultures, limited ability to identify rare pathogens if not present in the database, false susceptibility or resistance results, and incorrect MIC interpretation for certain antibiotics [101]. There are also more recent integrated, assembly-line style fully automated microbiology solutions, such as the BD Kiestra™ system (Becton Dickinson, Sparks, MD, USA) and the WASPLab^®^ system (Copan Diagnostics, Murrieta, CA, USA), which integrate plate inoculation, incubation, imaging, and MALDI-TOF MS interfacing, representing a higher level of automation [102]. The Biolog OmniLog System (Danaher Corporation, Washington, DC, USA) identifies bacteria based on metabolic activity rather than growth. It tests oxidation of multiple carbon sources using a tetrazolium-based redox dye, generating a metabolic fingerprint that is compared to a reference database. It can also be adapted for high-throughput, label-free applications, such as automated MIC determination [103].

### 6.2. Rapid Cartridge-Based Diagnostics

Rapid cartridge-based diagnostics are fully automated platforms that integrate sample preparation, nucleic acid extraction, amplification, and detection within single-use cartridges, providing fast, accurate results with minimal manual handling.

One of the most widely used cartridge-based platforms is the GeneXpert System (Cepheid, Sunnyvale, CA, USA). The GeneXpert MTB/RIF assay uses qPCR to detect *M. tuberculosis* complex and rifampicin resistance (*rpoB*) within two hours, with *rpoB* serving as a surrogate marker for multidrug-resistant tuberculosis. It is highly sensitive in smear-positive respiratory samples but less so in low-burden or extrapulmonary specimens [104]. Additional GeneXpert panels detect respiratory (*S. pyogenes*) and genital pathogens (*C. trachomatis*, *N. gonorrhoeae*, *S. agalactiae*), as well as healthcare-associated infection markers (MSSA/MRSA, carbapenemases KPC/NDM/VIM/OXA-48/IMP, *C. difficile,* including 027 strain, *vanA*), with most results available in ~30 min [105].

The BioFire FilmArray System (bioMérieux, Marcy-l’Étoile, France) is a multiplex PCR platform detecting pathogens from respiratory, gastrointestinal, and meningitis panels, as well as organisms and resistance genes from positive blood cultures. It delivers results in ~1 h, enabling rapid, targeted antimicrobial therapy in severe infections, improving clinical outcomes [106].

Abbott ID NOW (Abbott Laboratories, Abbott Park, IL, USA) is a rapid isothermal nucleic acid amplification platform for respiratory pathogens (e.g., *S. pyogenes*). Results are available in 5–15 min, making it suitable for clinics and emergency departments, though it does not provide AST [107].

All the above-mentioned platforms are valuable for point-of-care testing, but implementation is limited by cost in low-income settings. Limitations include reduced sensitivity in low-pathogen samples, restricted pathogen panels, and lack of full antimicrobial susceptibility data. As they detect nucleic acids rather than viable organisms, results must be interpreted in the clinical context.

## 7. Quality Control, Validation, and American Type Culture Collection Strains

Quality control (QC) is essential to ensure the accuracy and reproducibility of microbial identification and AST in microbiology laboratories. QC strains, also referred to as standard strains or reference strains, are routinely employed to validate culture media performance, biochemical and automated identification systems, disk diffusion and broth microdilution AST methods, detection of defined resistance phenotypes, and molecular diagnostic assays [108]. They are genetically stable, well characterized both phenotypically and genotypically, standardized, and widely accepted. They are traceable to authenticated source material, thereby enabling reproducibility and comparability of results across laboratories and geographic regions. Several internationally recognized culture collections maintain and distribute reference strains. Among the most widely used are the American Type Culture Collection (ATCC), the National Collection of Type Cultures (NCTC), the National Collection of Industrial, Food and Marine Bacteria (NCIMB), and the World Data Centre for Microorganisms (WDCM), which serves as a global database of registered culture collections [109,110]. ATCC strains are globally adopted and largely supported by performance standards established by the CLSI, which publishes validated zone diameter and MIC ranges for specific ATCC strains used in AST [108].

Table 4 and Table 5 represent the most commonly used ATCC strains and their application in routine diagnostic testing and research.

Testing QC strains at defined intervals enables early detection of methodological errors, reagent deterioration, or instrument malfunction, thereby ensuring compliance with accreditation standards and maintaining inter-laboratory comparability. ATCC strains are essential for implementing quality assurance through calibration and maintenance of automated systems and instruments, proficiency testing for controlling the fulfillment of standard operating procedures and personnel competency, and monitoring of supplies and the entire sample processing from admission in the laboratory through results reporting. Accredited laboratories use the international standard ISO 15189, which integrates these elements into a single quality management system for improved patient safety [121].

## 8. Current Trends and Future Perspectives

### 8.1. Peptide Nucleic Acid Fluorescence In Situ Hybridization

Peptide nucleic acid (PNA) fluorescence in situ hybridization (FISH) uses fluorescently labeled synthetic peptide nucleic acid probes to bind and detect species-specific bacterial rRNA directly in clinical specimens (e.g., blood cultures, tissues, and peritoneal fluid). This approach allows rapid identification of both Gram-positive (e.g., *Staphylococcus* spp. and *Enterococcus* spp.) and Gram-negative (e.g., *E. coli*, *K. pneumoniae*, and *P. aeruginosa*) pathogens in bloodstream infections [122,123]. Next-generation QuickFISH assays further reduce turnaround times to <30 min by eliminating washing steps [123]. Recent advances include Förster resonance energy transfer (FRET)-based PNA probes, which penetrate bacterial cells more efficiently, exhibit higher mismatch sensitivity, and can accurately distinguish multiple bacterial species common in bacteremia with 96–99.9% accuracy, which is crucial for timely treatment of acute infectious diseases like sepsis [124]. PNA-FISH has also demonstrated high accuracy in non-blood samples, including burn wound specimens [125]. PNA–FISH also shows high accuracy and is valuable in veterinary diagnostics, including rapid detection of *Mycobacterium bovis* in bovines and *Campylobacter* spp. in food samples [126,127].

The uncharged, stable peptide-like backbone of PNA probes confers superior bistability, stronger binding affinity, and better cell penetration compared to DNA probes, enabling rapid, sensitive, and specific hybridization [122]. However, PNA-FISH requires a fluorescence microscope and a minimum bacterial concentration of about 10^5^ CFU/mL for detection [122].

### 8.2. Volatile Organic Compound Profiling for Bacterial Identification

Volatile Organic Compounds (VOCs) have gained considerable attention due to their potential as non-invasive biomarkers for disease detection [128]. VOCs are a diverse group of carbon-based compounds, including aldehydes, ketones, alcohols, acids, amines, terpenes, and sulfides, which are volatile at ambient temperature and detectable in exhaled breath and biological fluids, such as urine, blood, feces, and sweat [129]. VOCs are mainly secondary products of bacterial fermentation [130]. Therefore, their composition depends on microbial species, strain, growth phase, environmental conditions, pH, and host–pathogen interactions, which makes them valuable as potential biomarkers [131]. Various microorganisms, including human pathogens, are known to produce characteristic volatile metabolites, which can be detected through headspace screening of bacterial cultures [132]. This enables in vitro identification and discrimination using VOC “fingerprinting” or “smell printing” approaches [133,134]. Since some VOCs are unique to certain pathogens, they can be used as biomarkers in the identification of specific bacteria. Furthermore, monitoring the profiles of microbial VOCs can enable the detection of emerging infections, which can in turn allow clinicians to implement antimicrobial therapy in a timely manner and monitor its effectiveness, both of which are fundamental components of personalized medicine.

Testing for volatile biomarkers offers an option for developing rapid and potentially inexpensive disease screening tools. The analysis of microbial VOCs in exhaled breath and human bodily fluids is a promising clinical approach, as it can enable the diagnosis of bacterial infections in a fast and non-invasive manner [135].

Two main strategies are used in VOC-based diagnostics: the first approach utilizes constantly present pathogen-specific biomarkers for detecting an infection in patients [136]. The second approach, which is based upon a combination of pathogen-specific biomarkers and those generated in vivo during reciprocal host–pathogen interactions, has been successfully utilized for TB detection [137,138,139].

A variety of analytical methods allow for the detection and identification of VOCs. Gas chromatography coupled with mass spectrometry (GC–MS) is the gold standard for VOC detection. GC–MS does possess large databases for the identification of substances and the capability of separating and unequivocally identifying compounds. Over the last two decades, there has been growing interest in the experimental and clinical application of spectrometric methods for the detection of bacterial VOCs for the diagnosis of a very large number of different diseases that are caused by respiratory, gastrointestinal, bloodstream, urinary, and genital infections, as well as surgical site and wound infections and infections in newborn infants [140].

The tool, however, is costly, time-consuming, and requires a trained operator. In addition, the data acquired from the GC-MS analysis can be intricate, making interpreting the results challenging [141]. The major current limitations of the VOC profiling approach are the extremely high costs of laboratory instrumentation, depending on appropriate VOC-detecting technologies, and the lack of standardized sample collection and preconcentration procedures (devices), which are essential for effective laboratory implementation [128]. The detection of VOCs of microbial origin is an alternative approach with the potential to be a robust, fast, and relatively low-cost point-of-care method for pathogen differentiation and identification [142].

### 8.3. Nanomaterials-Based Detection

The incorporation of nanomaterials into bacterial identification workflows represents a significant shift from culture-based diagnostics to rapid, highly sensitive analytical platforms. Nanomaterial-based systems (NBSs) exploit unique nanoscale properties, such as large surface area, localized surface plasmon resonance (LSPR), enzyme-like catalytic activity, enhanced electron transfer, and adjustable fluorescence, to convert biological recognition events (e.g., bacterial or nucleic acid binding) into strong, measurable signals [143]. In the last decade, the rapid progress in nanotechnology has allowed for the creation of biosensors that can detect whole bacterial cells and specific surface markers, toxins, or genetic material much faster than traditional culture-based methods [143]. Many NBSs are designed for point-of-care use, enabling on-site testing with near real-time results. Such decentralized diagnostics can improve decision-making, especially in urgent clinical situations or in settings with limited laboratory infrastructure [144].

Unlike molecular tests, such as PCR, many NBSs boost the signal using their own physical and/or chemical properties. For example, plasmonic nanoparticles can change color when they cluster together, electrochemical nanomaterials can strengthen electrical signals, and nanozymes can produce visible color changes without using natural enzymes [145]. Because the signal is amplified directly by the material itself, the overall testing process becomes simpler while still remaining highly sensitive. This makes these approaches especially useful for rapid screening, when quick results and minimal equipment are important.

Although these platforms improve speed and sensitivity, accurate identification still depends on probe specificity. Nanomaterials alone do not ensure precise taxonomy and should be used within validated, standardized identification strategies before being widely used in routine clinical practice.

(a) Noble Metal (Gold and Silver) Nanoparticles

Gold nanoparticles (AuNPs) are widely used in bacterial detection due to their LSPR, a property that produces a visible color change upon particle aggregation [145]. The reason for the simple color shift in multiple rapid colorimetric tests and lateral flow assays lies in their underlying detection mechanism. When they are used for detection purposes, AuNPs are often coated with antibodies, aptamers, or DNA probes that recognize specific bacterial markers. At the moment when binding occurs, the nanoparticles either cluster or separate, leading to a measurable color change [143]. AuNP-based systems are sensitive and rapid, but a major limitation is that, in many cases, they can only differentiate targets at the genus or species level. This becomes especially difficult when trying to distinguish closely related strains, because many AuNP platforms lack the resolution needed for reliable identification at the strain level [127].

Silver nanoparticles (AgNPs) are often used in surface-enhanced Raman scattering (SERS) systems, where they strengthen Raman signals and help generate spectral fingerprints of bacteria [146]. These fingerprints can be related to differences in cell wall composition, membrane structures, or intracellular biomolecules, which allows for differentiation between bacterial species. Although this method can potentially allow more detailed differentiation, its accuracy depends on the method of substrate preparation and reliable reference spectra.

(b) Magnetic nanoparticles (MNPs)

Magnetic nanoparticles are commonly used to isolate and concentrate bacteria prior to the actual detection step. When coated with antibodies or other molecules, they can selectively bind bacterial cells and allow their rapid separation using a magnetic field. This approach makes it possible to recover bacteria from complex samples, such as blood, food products, or environmental matrices, helping to increase sensitivity and reduce background interference [144].

In many identification workflows, magnetic enrichment is combined with downstream techniques, such as PCR, fluorescence-based assays, or electrochemical detection [147]. While this combined strategy can improve total specificity and detection limits, the final performance often depends on how well the nanoparticles are functionalized and how efficiently they capture the target bacteria.

(c) Quantum Dots and Fluorescent Nanomaterials

Quantum dots (QDs), carbon dots (CDs), and upconversion nanoparticles (UCNPs) are increasingly used in bacterial detection because they produce strong and stable fluorescent signals. Unlike traditional fluorescent dyes, these materials are less prone to fading and can be tuned to emit light at different wavelengths. This makes them especially useful when more than one bacterium needs to be detected at the same time [143,148].

These systems work by attaching fluorescent nanoparticles to antibodies, aptamers, or DNA probes. When they bind to a specific bacterial cell or genetic sequence, the fluorescence changes in a measurable way. Because different nanoparticles can emit different colors, it becomes possible to detect multiple species within a single test [148]. When highly specific probes are used, these platforms can achieve species-level identification, and, in some cases, even approach strain-level differentiation. However, their performance can be affected by background signals in complex samples like blood or food [144].

(d) Electrochemical and nanozyme-based systems

Electrochemical biosensors based on nanomaterials such as graphene, carbon nanotubes, metal–organic frameworks (MOFs), and other conductive nanostructures can convert biological interactions directly into electrical signals. These systems are often fast, sensitive, and suitable for miniaturized or portable devices. When a bacterial target binds to the sensor surface, measurable changes in current, voltage, or impedance occur, allowing detection within a relatively short time frame [144,147].

Nanozymes are synthetic nanomaterials that mimic enzyme activity (e.g., peroxidase or oxidase), generating detectable signals without biological enzymes [145]. They are more stable, cost-effective, and less sensitive to environmental conditions than natural enzymes [149].

#### Standardization Challenges in Nanomaterial-Based Identification

Although nanomaterial-based systems enable rapid and sensitive bacterial detection, their routine use is limited by a lack of standardization in nanoparticle preparation, calibration, and signal interpretation. Reproducibility in complex samples (e.g., blood, food, and environmental matrices) remains challenging due to background interference, highlighting the need for improved harmonization and validation before clinical implementation.

### 8.4. Phage-Based Methods for Bacterial Identification

Phage-based methods (PBMs) are diagnostic techniques used to detect, identify, or differentiate bacterial species or strains based on bacteriophage–host specificity or phage-mediated reactions. They leverage the inherent specificity of bacteriophages for their bacterial hosts, enabling rapid, cost-effective in vitro detection of viable bacteria—even within complex samples—often bypassing the need for extensive culture. Using bacteriophages as diagnostic tools shows potential in a wide range of fields, including medicine, food safety, and agriculture [150]. Phages have been employed for decades, with typing schemes established for many clinically significant microorganisms, including *M. tuberculosis*, *Enterococci*, *Salmonella*, *Clostridium*, *Listeria*, and toxigenic *E. coli*, among others [151]. Despite their long history, few phage-based diagnostics have entered routine clinical practice. Adoption has been limited by regulatory and validation hurdles, the need for standardized preparations, limited commercial availability, competition from established methods (culture, PCR, etc.), narrow host specificity, and the logistical challenges of handling live viral reagents [152]. Nevertheless, PBMs, such as phage amplification, reporter phages, phage labelling, and phage capture elements, have the potential to directly detect pathogens in various clinical samples, thereby eliminating the need for primary culture.

Key PBM approaches broadly fall into and include phage typing, reporter phages, phage amplification assays, and phage capture/immobilization, as shown in Table 6.

## 9. Artificial Intelligence in Identification and Management of Bacterial Infections

The increasing digitization of microbiology laboratories has generated datasets of unprecedented scale and complexity. WGS, metagenomics, high-resolution mass spectrometry, and automated microscopy produce vast quantities of structured and unstructured data that exceed the capacity of conventional analytical approaches. Artificial intelligence (AI), particularly machine learning (ML) and deep learning (DL), has, therefore, emerged as a transformative tool in bacterial identification and infection management. However, AI promises enhanced speed and precision, but its integration into routine practice raises a critical question: the occurrence of meaningful progress without rigorous standardization. AI-based systems significantly accelerate bacterial identification workflows. Unlike conventional culture-based diagnostics, ML algorithms can analyze complex datasets within minutes, enabling real-time or near real-time insights. As emphasized by Topol, AI in medicine is most effective when it augments human expertise rather than replaces it, enabling higher-performance decision-making, especially in time-sensitive conditions like sepsis [161].

AI enhances MALDI-TOF MS proteomics by predicting resistotypes from complex spectral patterns beyond conventional similarity scoring [162]. However, reproducibility remains a concern, as spectral variability from instruments, sample preparation, or database updates can cause inconsistent AI-enhanced MALDI-TOF results across labs.

Advanced AI in microbiology leverages genomic and metagenomic data using DL and ML models to predict antibiotic resistance (e.g., identify key mutations in *M. tuberculosis*) directly from sequences, enabling rapid genotype-to-phenotype predictions and reducing reliance on lengthy phenotypic testing [163,164].

AI also aids image-based diagnostics, with convolutional neural networks classifying bacterial morphology from microscopy images. While useful for rapid or high-throughput screening, performance depends on standardized imaging and curated training data, as variability in staining, hardware, or lighting can reduce robustness [165,166]. Beyond laboratory diagnostics, AI predictive models trained on electronic health records (EHRs) can identify patients at risk of bloodstream infection, forecast sepsis progression, and estimate local resistance patterns, enabling optimized empirical therapy [167]. Additionally, AI-driven analysis of large-scale microbiological surveillance data can identify outbreak strains, detect clusters, and map transmission pathways. However, these models often rely on institution-specific datasets, limiting generalizability.

Despite these advances, genomic AI tools rely on high-quality, representative datasets; models trained on geographically restricted data may fail to generalize well beyond specific institutions and regions. As Wiens and Shenoy noted, ML in healthcare is particularly vulnerable to dataset shift, where differences between training and real-world data degrade model performance [168]. In microbiology, this can stem from variations in sequencing platforms, library preparation protocols, or local strain epidemiology. Without harmonized data standards, multicenter validation, and transparent reporting, as emphasized by Esteva et al., AI predictions risk remaining context-dependent rather than broadly reliable [169]. Another key limitation is interpretability: many DL systems act as “black boxes,” offering predictions without a clear rationale. Explainable AI is essential for clinician trust, regulatory approval, and professional acceptance. Secure, privacy-protected, and representative datasets, along with standardized frameworks for sharing, annotation, and benchmarking, are critical. AI should complement human expertise, so-called collaborative intelligence, supported by clear performance metrics and harmonized validation [161]. While AI can accelerate bacterial identification and infection management, its reliability depends on consistent data, transparent validation, and reproducible, interpretable frameworks for global application.

## 10. Differences and Setting-Specific Specificities in Bacterial Identification

In the last decade, bacterial identification practices across clinical, veterinary, food, and public health laboratories have evolved rapidly, reflecting advances in analytical technologies, increasing diagnostic demands, and global efforts to harmonize laboratory procedures. Although each environment runs under distinct regulatory and operational constraints, several cross-cutting trends have reshaped microbial diagnostics across sectors [170,171].

Culture remains central to bacterial identification, providing pure isolates for antimicrobial susceptibility testing, essential in clinical microbiology.

One of the most influential advances in bacterial identification is the widespread adoption of MALDI-TOF MS, now the dominant method in clinical microbiology due to its speed, accuracy, and cost-effectiveness [170,172]. Veterinary laboratories, traditionally more heterogeneous, are increasingly adopting MALDI-TOF, with over 40% now using it routinely according to a 2024 European survey, reflecting a shift toward faster, more standardized identification workflows [171]. In the area of food microbiology, adoption is slower but steadily increasing, particularly in reference laboratories responsible for confirming foodborne pathogens [173,174].

**Figure 5:**
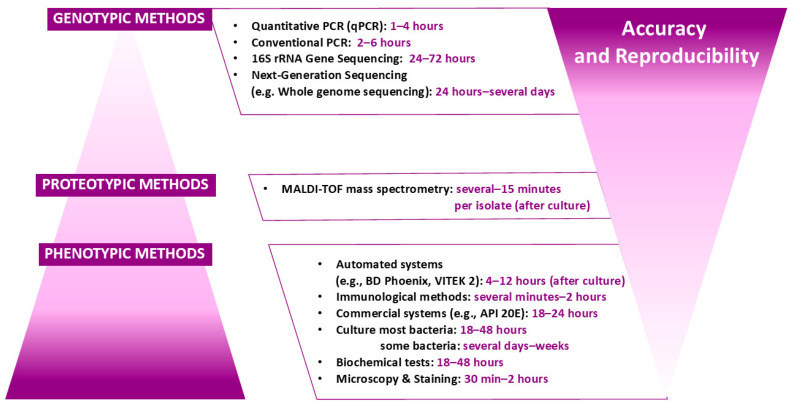
Comparative turnaround times for bacterial identification methods.

Molecular diagnostics have expanded significantly across all environments, offering high sensitivity and specificity even in low-burden or uncultivable samples and can detect resistance or virulence genes, but require specialized equipment and expertise. PCR and qPCR remain foundational tools, but the trend has shifted toward multiplex PCR assays capable of detecting multiple pathogens simultaneously, improving diagnostic efficiency in both clinical and veterinary settings [59,175]. In food laboratories, PCR has become indispensable for confirming priority pathogens such as *Salmonella*, L. *monocytogenes*, and Shiga toxin-producing *E. coli* [176,177], supported by updated ISO and FDA BAM methods (e.g., ISO 6579-1:2020; FDA BAM Chapter 5, 2022) [178,179]. The COVID-19 pandemic accelerated the adoption of rapid molecular assays, particularly isothermal amplification methods such as LAMP, which gained traction in point-of-care clinical settings and field-based veterinary diagnostics [180,181]. Turnaround times, accuracy, and reproducibility for various bacterial identification methods are shown in Figure 5.

Automation is another defining trend. Clinical laboratories increasingly rely on automated incubation systems, digital colony counters, and integrated antimicrobial susceptibility testing platforms to address workforce shortages and improve turnaround times [102]. Veterinary and food laboratories are adopting automation more gradually, but digital colony analysis and automated enrichment systems are becoming more common in high-throughput environments [173,182].

Nowadays, WGS and metagenomics have transitioned from research tools to routine applications in public health and reference laboratories. WGS is now central to outbreak investigation, source attribution, and antimicrobial resistance profiling across human, animal, and food sectors [183]. Metagenomics, while still resource-intensive, is increasingly used for complex matrices such as environmental samples or food [184].

Artificial intelligence has appeared to be a promising, though still developing, part of bacterial identification. AI-assisted colony recognition, automated interpretation of chemolysis, and prediction of antimicrobial resistance from genomic data demonstrate strong potential across laboratory types [185].

Finally, the period is marked by strong international efforts toward harmonization. Updated EUCAST and CLSI guidelines, ISO standards, and coordinated veterinary laboratory initiatives have contributed to greater methodological consistency across sectors. Collectively, these trends illustrate a decisive movement toward faster, more precise, and more standardized bacterial identification across diverse laboratory environments.

**Figure 6:**
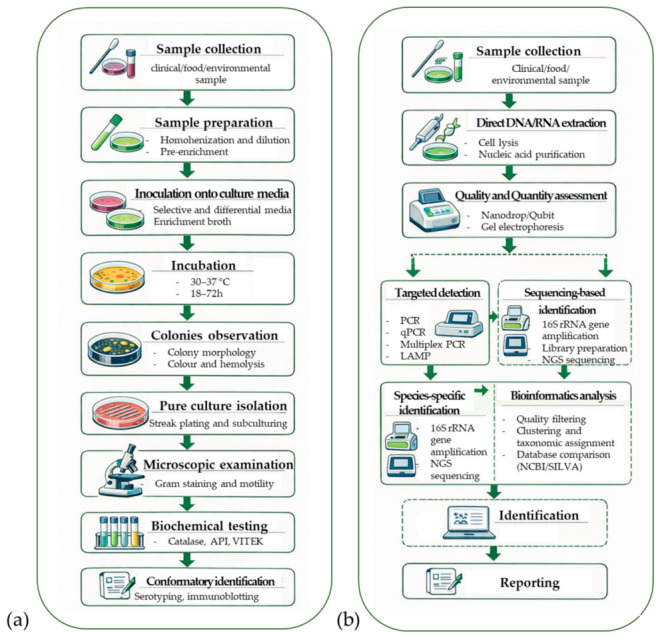
Workflow of bacterial identification approaches: (**a**) conventional methods, including culture-based, microscopic, biochemical, and serological approaches, and (**b**) modern approaches, including molecular methods, whole-genome sequencing, and metagenomics.

A summary of methods and their sequential application in bacterial identification is displayed in Figure 6.

## 11. Conclusions and Future Directions

Bacterial identification remains fundamental across clinical, veterinary, agricultural, and research settings. While conventional methods retain a foundational role, modern proteomic, molecular, and sequencing-based approaches have markedly improved speed, accuracy, and resolution, underscoring the importance of selecting appropriate methods based on application and setting. Emerging technologies, including CRISPR-, nanomaterial-, and phage-based platforms, as well as AI, further enhance diagnostic capabilities and enable rapid detection of challenging pathogens, with expanding clinical and industrial potential.

However, the transition toward integrated, high-throughput identification systems and digital innovation, which are transforming laboratory operations, is hindered by persistent challenges in standardization, validation, and accessibility, particularly for advanced approaches, such as WGS. Harmonization of methodologies, including bioinformatics frameworks and interpretative criteria, is essential to ensure reproducibility and comparability of results. Future efforts should prioritize the integration of innovative technologies into routine workflows alongside robust QC systems. Ultimately, modernization must be coupled with rigorous standardization to ensure reliable, accurate, and clinically meaningful bacterial identification and to support consistent AST across settings.

## Figures and Tables

**Table 1:** Growth requirements and incubation timeframes for bacterial cultivation [16].

**Category**	**Growth Requirements**	**Examples**
Obligate aerobes	Require atmospheric O_2_ (~21% O_2_)	*Mycobacterium tuberculosis*
Facultative anaerobes	Grow with or without O_2_	*Escherichia coli*
Obligate anaerobes	Grow only in the absence of O_2_ (≈0% O_2_)	*Clostridioides difficile*
Capnophilic bacteria	Require increased CO_2_ (5–10%)	*Neisseria gonorrhoeae*, *Haemophilus influenzae*
Microaerophilic bacteria	Reduced O_2_ (2–10% O_2_) + increased CO_2_ (5–10%)	*Campylobacter jejuni*
**Category**	**Temperature**	**Examples**
Mesophiles	Optimal growth at 20–45 °C	Most human pathogens (37 °C), *Mycobacterium marinum* (25–30 °C)
Psychrophiles	Typically ≤15 °C	*Listeria monocytogenes* (4–37 °C)
Thermophiles	Typically ≥45 °C, often 50–70 °C	*Campylobacter jejuni* (42 °C)
**Category**	**Incubation time**	**Examples**
Fast-growing	18–24 h	Common bacteria (*Staphylococcus* spp., *Escherichia coli*)
Moderately fast-growing	48 h	*Neisseria* spp., *Campylobacter* spp.
Slow-growing	~2–7 days	*Legionella pneumophila*, *Brucella* spp., *Nocardia* spp.
Slow-growing	≥2–8 weeks	*Mycobacterium tuberculosis*, *Mycobacterium avium complex*

**Table 2:** Biochemical reactions of commonly isolated bacteria [17].

Species	Catalase	Citrate	Gas	H_2_S	Indole	Motility	Methyl Red	Nitrate	Oxidase	Spores	Urease	Voges Proskauer	Glucose	Lactose
*Acinetobacter baumannii*	+	+	-	−	−	−	−	−	−	−	−	−	+	m− s+
*Actinomyces israelii*	NA	NA	NA	+	−	−	v	v	NA	NA	−	−	+	+
*Aeromonas caviae*	+	+	−	−	+	v	NA	+	+	−	−	−	−	v
*Aeromonas hydrophila*	+	+	+	+	+	+	NA	+	+	−	−	+	+	v
*Alcaligenes faecalis* subsp. *faecalis*	+	+	NA	−	−	+	NA	−	+	−	−	NA	−	NA
*Bacillus anthracis*	+	NA	−	NA	+	−	NA	+	−	+	−	+	+	−
*Bacillus cereus*	+	+	NA	NA	−	+	−	v	−	+	v	+	+	−
*Bacillus subtilis*	+	+	−	NA	−	+	−	+	v	+	−	+	+	v
*Bacteroides fragilis*	+	NA	NA	NA	v	−	NA	−	v	−	−	NA	+	+
*Bifidobacterium bifidum*	−	NA	−	NA	−	−	NA	−	−	−	NA	NA	−	−
*Bordetella pertussis*	+	−	NA	NA	NA	−	NA	−	+	−	−	NA	−	−
*Brucella melitensis*	+	NA	NA	−	−	−	−	+	+	−	+	−	+	NA
*Burkholderia cepacia*	+	+	NA	NA	NA	+	NA	−	v	-	+	NA	NA	−
*Burkholderia pseudomallei*	+	m−	−	−	-	+	NA	+	+	−	-	NA	+	−
*Campylobacter fetus* subsp. *fetus*	+	NA	NA	-	NA	+	NA	+	+	−	−	NA	+	−
*Campylobacter jejuni*	+	NA	NA	−	NA	+	NA	+	+	−	−	NA	−	NA
*Chlamydia trachomatis*	NA	NA	NA	NA	NA	−	NA	NA	NA	NA	NA	NA	NA	NA
*Citrobacter freundii*	+	+	+	+	−	+	+	+	−	−	v	−	NA	+
*Clostridium botulinum*	−	NA	NA	+	−	m+	NA	NA	−	+	NA	NA	+	−
*Clostridium difficile*	−	NA	NA	+	−	+	NA	−	−	+	−	NA	+	−
*Clostridium perfringens*	−	NA	+	+	−	−	NA	v	−	+	NA	NA	+	+
*Clostridium tetani*	−	NA	+	+	v	+	NA	−	NA	+	NA	NA	−	−
*Corynebacterium diphtheriae*	+	−	v	+	−	−	+	+	−	−	−	NA	+	−
*Cronobacter sakazakii*	+	+	+	−	−	+	−	+	−	−	−	+	+	+
*Edwardsiella tarda*	+	−	+	+	+	+	+	+	−	NA	−	−	+	−
*Enterobacter aerogenes*	+	+	+	−	−	+	−	+	−	−	−	+	+	+
*Enterobacter cloacae*	+	+	+	−	−	+	−	+	−	−	−	+	+	+
*Enterococcus faecalis*	−	−	NA	−	−	−	NA	NA	−	−	−	+	+	+
*Enterococcus faecium*	−	−	NA	−	NA	−	NA	NA	NA	−	NA	−	+	+
*Escherichia coli*	+	−	+	−	+	+	+	+	−	−	−	−	+	+
*Francisella tularensis* subsp. *tularensis*	+	NA	−	+	−	−	NA	−	−	−	−	NA	+	−
*Fusobacterium necrophorum*	−	NA	+	+	+	−	−	−	−	−	NA	−	v	−
*Gardnerella vaginalis*	−	NA	−	−	−	−	+	−	−	−	−	−	+	−
*Haemophilus aegyptius*	+	NA	−	−	−	−	NA	−	+	NA	+	NA	+	−
*Haemophilus influenzae*	+	NA	−	−	v	−	NA	+	+	NA	v	NA	+	−
*Haemophilus parainfluenzae*	v	NA	v	+	v	−	NA	+	+	NA	v	NA	+	−
*Hafnia alvei*	+	+	+	−	−	+	v	+	−	−	−	+	+	−
*Helicobacter pylori*	+	NA	NA	+	NA	+	NA	−	+	−	+	NA	+	NA
*Kingella kingae*	−	−	−	v	−	−	NA	−	+	−	−	NA	+	−
*Klebsiella granulomatis*	+	+	NA	−	−	−	−	−	+	NA	+	+	+	+
*Klebsiella oxytoca*	+	+	v	−	+	−	−	+	−	−	+	+	+	+
*Klebsiella pneumoniae*	+	+	+	−	−	−	−	+	−	−	+	+	+	+
*Lactobacillus* spp.	−	−	−	−	−	m−	m− s+	−	−	−	−	−	+	+
*Listeria monocytogenes*	+	NA	−	−	−	+	+	−	−	−	−	+	+	+
*Morganella morganii* subsp. *morganii*	+	−	+	−	+	+	+	+	−	−	+	−	+	−
*Mycobacterium tuberculosis*	−	NA	NA	NA	NA	NA	NA	+	NA	NA	+	NA	NA	NA
*Neisseria gonorrhoeae*	+	NA	−	−	NA	NA	NA	−	+	NA	NA	NA	+	−
*Pasteurella multocida*	+	−	−	−	+	−	−	+	+	−	+	−	+	−
*Proteus mirabilis*	+	+	+	+	−	+	+	+	−	−	+	−	+	−
*Providencia stuartii*	+	+	−	−	+	+	+	+	−	−	−	−	+	−
*Pseudomonas aeruginosa*	+	+	−	−	−	+	−	+	+	−	−	−	−	−
*Salmonella Typhi*	+	−	−	+	−	+	+	+	−	−	−	−	+	−
*Serratia marcescens*	+	+	v	−	−	+	−	+	−	−	+	+	+	−
*Shigella flexneri*	+	−	+	−	v	−	+	+	−	−	−	−	NA	−
*Staphylococcus aureus*	+	+	−	−	−	−	+	+	−	−	+	+	+	+
*Staphylococcus epidermidis*	+	−	+	+	NA	−	−	+	−	NA	+	+	+	+
*Streptococcus agalactiae*	−	NA	NA	NA	NA	+	NA	NA	−	−	−	v	+	v
*Streptococcus canis*	−	NA	NA	NA	NA	NA	NA	NA	NA	NA	−	−	+	+
*Streptococcus mutans*	−	NA	NA	NA	NA	−	NA	NA	−	−	−	+	+	+
*Streptococcus pneumoniae*	−	NA	NA	NA	NA	−	NA	NA	−	−	−	−	+	+
*Streptococcus pyogenes*	−	NA	NA	NA	NA	−	NA	NA	NA	−	−	−	+	+
*Vibrio cholerae*	NA	+	−	−	+	+	−	+	+	−	−	v	+	v
*Yersinia pestis*	+	−	−	−	−	−	+	+	−	−	−	−	+	−

+: positive; −: negative; NA: not applicable; v: variable; m: most; s: some.

**Table 3:** Comparison of target, advantages, and limitations of agglutination-based assays.

Test	Target/Application	Commercial Diagnostic Kits and Manufacturer	Advantages	Limitations
Latex agglutination	Identification of bacterial antigens in patient specimens or cultured colonies: - Meningitis pathogens in CSF (*Streptococcus* group B, *Haemophilus influenzae* type b, *Streptococcus pneumoniae*, *Neisseria meningitidis* groups A, B, C, Y, W135, *E. coli* K1). - Beta-hemolytic *Streptococcus* A–G - Thermophilic *Campylobacter* spp. (*C. jejuni*, *C. coli*, *C. lari*). - Streptococcal grouping kit. - Beta-hemolytic *Streptococcus* A, B, C, D, F, G. - *Staphylococcus*.	- Wellcogen™ Bacterial Antigen Test Kit Instructions for Use; Thermo Fisher Scientific: Waltham, MA, USA, https://www.thermofisher.com (accessed 7 February 2026). - Remel™ PathoDX™ Streptococcal Grouping Latex Kit Instructions for Use; Thermo Fisher Scientific: Waltham, MA, USA, https://www.thermofisher.com (accessed 7 February 2026). - Streptex™ Streptococcal Grouping Kit Instructions for Use; Thermo Fisher Scientific: Waltham, MA, USA, https://www.thermofisher.com (accessed 7 February 2026). - BactiStaph™ Latex Test Kit Instructions for Use; Thermo Fisher Scientific: Waltham, MA, USA, https://www.thermofisher.com (accessed 7 February 2026).	Rapid, visible clumping, low-cost, minimal equipment, point-of-care use.	Operator-dependent, visual subjectivity, lower sensitivity than ELISA, prozone/postzone effects.
Slide agglutination	- Detection of coagulase and protein A of *Staphylococcus aureus* - *Salmonella/Brucella* antisera (paper-based assays): serotyping reagents.	- Staphaurex™ Latex Agglutination Test Instructions for Use; Thermo Fisher Scientific: Waltham, MA, USA, https://www.thermofisher.com (accessed 7 February 2026). - BD Difco™ Salmonella O and H Antisera; BD: Franklin Lakes, NJ, USA. Product page (accessed 7 February 2026).	Simple, rapid, suitable for non-technical end-user, open reading time.	Requires fresh colonies, subjective reading, short reading time, operator-dependent, evolving technique.
Coagglutination	- Pneumococcal serotyping. - *Vibrio cholerae* O1/O139 detection.	- ImmuLex™ Pneumotest Kit Instructions for Use; Statens Serum Institute: Copenhagen, Denmark, https://ssidiagnostica.com/solutions/antisera/pneumococcus-antisera/immulex-pneumococcus-kits/ (accessed 5 January 2026). - BengalScreen Coagglutination Test Instructions (research protocol); University of Maryland: College Park, USA, https://pubmed.ncbi.nlm.nih.gov/8576349/ (accessed 5 January 2026).	High specificity and sensitivity, direct use on colonies or clinical samples.	Requires cultivation and trained operator, careful reagent storage, time-consuming, potential false negatives.
TPHA (*Treponema pallidum* haemagglutination)	- Serological detection of *Treponema pallidum* antibodies in serum.	- IMMUTREP^®^ TPHA (*Treponema pallidum* haemagglutination) Kit Instructions for Use; Omega Diagnostics Ltd.: Alloa, Scotland, UK, https://www.omegadx.com/Portals/0/TPHA_MSDS.pdf (accessed 20 January 2026).	High sensitivity and specificity, simple to perform, widely used in diagnostic labs.	Cannot distinguish active vs. past infection; serum required, may need confirmatory testing.

**Table 4:** Typical ATCC strains and their key biochemical traits.

Bacterial Species	ATCC No.	Key Biochemical Traits	References
*Enterococcus faecalis*	29212	Catalase –; bile esculin +; growth in 6.5% NaCl +; PYR +	[111]
*Streptococcus pneumoniae*	49619	Alpha hemolysis; catalase –; optochin sensitive; bile solubility +	[111]
*Staphylococcus aureus*	25923	Catalase +; coagulase +; mannitol fermentation +; oxidase –; beta-hemolytic	[17]
*Staphylococcus aureus*	29213	Catalase +; coagulase +; mannitol fermentation +; oxidase –; beta-hemolytic	[17]
*Staphylococcus epidermidis*	12228	Catalase +; coagulase –; novobiocin sensitive; nonhemolytic	[17]
*Escherichia coli*	25922	Lactose +; indole +; MR +; VP –; citrate –; oxidase –; catalase +; gas from glucose +	[111]
*Klebsiella pneumoniae*	700603	Nonmotile; lactose fermenter; citrate +; VP +; indole –; urease +	[111]
*Pseudomonas aeruginosa*	27853	Oxidase +; catalase +; lactose –; nitrate reduction +; motile; pigment (pyocyanin) often produced	[111]
*Acinetobacter baumannii*	19606	Oxidase –; non-fermenter; nonmotile; lactose –	[111]
*Salmonella enterica*	14028	H_2_S +; lactose –; indole –; citrate +; lysine decarboxylase +	[111]
*Shigella flexneri*	12022	Nonmotile; catalase +; glucose +; gas from glucose −; MR +; VP −; citrate −; urease −	[111]
*Campylobacter jejuni*	33560	Microaerophilic; oxidase +; hippurate +; motile	[111]
*Clostridioides difficile*	9689	Anaerobe; catalase –; characteristic toxin production	[17]
*Haemophilus influenzae*	49247	Nonmotile; microaerophilic; catalase +; oxidase +; require NAD & hemin	[111]
*Neisseria gonorrhoeae* (penicillin resistant)	49226	Catalase +; oxidase +; glucose +; maltose −; lactose −	[111]
*Listeria monocytogenes*	19115	Catalase +; oxidase −; hippurate +; CAMP + (weakly); motile at 25 °C; beta-hemolytic	[17]
*Mycobacterium tuberculosis* (isoniazid and rifampicin susceptible)	27294	Niacin +; nitrate reduction +	[111]

**Table 5:** Application of resistant ATCC strains in various quality control analyses.

Bacterial Species	ATCC No.	Quality Control Purpose	References
ANTIMICROBIAL RESISTANCE			
*Escherichia coli*	35218	β-lactamase production, food microbiology, biofilm testing	[108,112]
*Klebsiella pneumoniae*	700603	ESBL production, biofilm analysis, bacteriophage testing, nanoparticle activity	[108,113]
*Enterococcus faecalis*	51299	Vancomycin-resistance, interbacterial relations and activity, pathogenesis	[114,115]
*Staphylococcus aureus*	43300	MRSA, inter-microbial relations, antimicrobial and antibiofilm activity	[108,116]
FASTIDIOUS BACTERIA			
*Haemophilus influenzae*	49247	AST (HTM media)	[108,117]
*Neisseria gonnorhoeae*	49226	AST (GC media)	[108,118]
*Campylobacter jejuni*	33560	Microaerophilic growth	[119]
ANAEROBES			
*Bacteroides fragilis*	25285	Anaerobic AST	[108]
*Clostridioides difficile*	700057	Anaerobic growth/toxin	[120]

**Table 6:** Comparison of phage-based identification methods.

Method	Principle	Detection Type	Advantages	Limitations	References
Phage Typing	Lytic phages infect unknown bacterial lawns; plaques reveal species/strains	Plaques	Simple, well-established; can differentiate closely related pathogens	Slow, requires culture	[153]
Reporter Phages	Engineered phages deliver reporter genes (e.g., luciferase, fluorescent proteins) into viable bacteria	Fluorescence/ luminescence	Rapid, specific; detects viable bacteria only	Requires genetic modification	[154]
Phage Amplification Assay	Phages infect and lyse host; progeny phages or intracellular markers detected	Plaques/PCR	Highly sensitive; enhances specificity via PCR	Multi-step, requires culture	[151,155,156,157]
Capture/Biosensor	Phage components (tail fibers, endolysins) selectively bind bacteria; captured cells detected by culture, ELISA, or qPCR; can use functionalized surfaces for label-free detection	qPCR/ELISA/ sensors	Direct, rapid, specific; label-free detection possible	Equipment-dependent	[158,159]
Phage Display	Foreign peptides or proteins fused to phage coat proteins for high-throughput screening of target-binding molecules	Binding assays	High-throughput; identifies immunogenic or target-binding proteins	Not direct detection of bacteria	[160]

## Data Availability

The original contributions presented in this study are included in the article/Supplementary Material. Further inquiries can be directed to the corresponding author.

## Supplementary Materials

The following supporting information can be downloaded at: https://www.mdpi.com/article/10.3390/ijms27115092/s1.

## Abbreviations

The following abbreviations are used in this manuscript:

AMR	Antimicrobial resistance
WHO	World Health Organization
PCR	Polymerase chain reaction
AST	Antimicrobial susceptibility testing
CLSI	Clinical and Laboratory Standards Institute
EUCAST	European Committee on Antimicrobial Susceptibility Testing
CAP	Community-acquired pneumonia
TSI	Triple sugar iron
API	Analytical profile index
ELISA	Enzyme-linked immunosorbent assay
FCM	Flow cytometry
UAT	Urinary antigen testing
PCVs	Pneumococcal conjugate vaccines
LFI	Lateral flow immunoassay
MALDI-TOF MS	Matrix-Assisted Laser Desorption Ionization–Time of Flight Mass Spectrometry
FTIE	Fourier transform–infrared
RFLP	Restriction fragment length polymorphism
MLST	Multilocus sequence type
TGS	Third-generation sequencing
NGS	Next-generation sequencing
MICs	Minimum inhibitory concentrations
ATCC	American Type Culture Collection
NCTC	National Collection of Type Cultures
NCIMB	National Collection of Industrial, Food, and Marine Bacteria
WDCM	World Data Centre for Microorganisms
VOCs	Volatile Organic Compounds
NBSs	Nanomaterial-based systems
LSPR	Localized surface plasmon resonance
SERS	Surface-enhanced Raman scattering
MOFs	Metal–organic frameworks
PBMs	Phage-based methods
AI	Artificial intelligence
ML	Machine learning
DL	Deep learning
