# Supplementary material for: Evolving Approaches to Bacterial Identification: A Review of Classical and Modern Techniques
Source: Int J Mol Sci. 2026 Jun 4;27(11):5092. doi: 10.3390/ijms27115092 (PMC13256776; doi:10.3390/ijms27115092)
Supplement: Supplementary file 1 [file ijms-27-05092-s001.zip › Supplementary Table S1.pdf]

Supplementary Table S1. Commonly used solid bacterial culture media and their properties [12]

Media name	Type	Purpose	Key components	Interpretation (examples)
**Nutrient agar**	N	General cultivation	Peptone, beef extract	Growth of non-fastidious bacteria
**Blood agar**	E/D	Growth of fastidious organisms; hemolysis detection	5% sheep or horse blood	- α hemolysis (green): *Streptococcus pneumoniae* and viridans streptococci;<br>- β hemolysis (clear/yellow): *Streptococcus pyogenes, S. agalactiae*;<br>- γ (no hemolysis): *Enterococcus* spp.
**Chocolate agar**	E	Growth of delicate, fastidious bacteria	Partially hemolyzed blood or hemoglobin powder (source of factor X-haemin)	*Haemophilus* spp., *Neisseria* spp.
**Campy blood agar**	S	Inhibits: *Enterobacteriaceae*, staphylococci, yeast; enriches: *Campylobacter* spp.	Cephalothin, amphotericin B, trimethoprim, vancomycin, polymyxin B (IA)	*Campylobacter* spp. (42 °C favors *Campylobacter jejuni*)
**Mannitol-salt agar**	S/D	Inhibits: Gram-negative and Gram-positive bacteria other than *Staphylococcus* spp.; enriches: *Staphylococcus aureus*	NaCl (IA); mannitol, phenol red (DA) - yellow in acidic conditions	*S. aureus* (ferments mannitol): yellow colonies on yellow medium;<br>Coagulase negative staphylococci (do not ferment mannitol): pink colonies on red medium
**MacConkey agar**	S/D	Inhibits: Gram-positive bacteria; enriches: Gram-negative enteric bacteria	Bile salts, crystal violet (IA); lactose, neutral red (I) - turns pink/red in acidic conditions	Lactose fermenters (*Escherichia coli*, *Klebsiella* spp.): pink colonies;<br>Non-lactose fermenters (*Salmonella* spp., *Proteus* spp., *Yersinia* spp., *Pseudomonas* spp.): colorless
**Eosin Methylene Blue (EMB) agar**	S/D	Inhibits: Gram-positive bacteria; enriches: Gram-negative bacteria	Eosin Y and methylene blue (IA and I) - dark purple or metallic green in acidic conditions, lactose, sucrose (DA)	Strong lactose fermenters (*E. coli*): blue-black colonies with green metallic sheen;<br>Weak fermenters (*Enterobacter aerogenes*): purple;<br>Non-fermenters (*Pseudomonas* spp.): colorless
**Xylose Lysine Deoxycholate (XLD) agar**	S/D	Inhibits: Gram-positive bacteria; enriches: enteric pathogens (*Salmonella* spp., *Shigella* spp.)	Bile salts, NaCl (IA); lactose, sucrose, xylose, phenol red (DA); sodium thiosulfate, ferric ammonium citrate for H_2S production (DA)	*Salmonella* spp. - red with black center (xylose ferment, lysine decarboxylation, H_2S); *Shigella* spp. - red (no xylose fermentation or H_2S); *E. coli* - yellow (lactose/sucrose ferment, no lysine decarboxylation).
**Cefsulodin Irgasan Novobiocin (CIN) agar**	S	Inhibits: most bacteria<br>Enriches: *Yersinia* spp., *Aeromonas* spp.	Cefsulodin, irgasan, novobiocin (IA), neutral red (I)	Manitol fermenters (*Yersinia enterocolitica* and *Aeromonas* spp.): colonies with red center and transparent margin („bull's-eye"); incubation at 25°C for 48h

***Salmonella-Shigella* (SS) agar**	S/D	Inhibits: Gram-positive bacteria and most coliforms; enriches: enteric pathogens (*Salmonella* spp., *Shigella* spp.)	Bile salts, brilliant green (IA); lactose, neutral red (I); sodium thiosulfate, ferric ammonium citrate for H_2S production (DA)	Non-lactose fermenters. *Salmonella* spp.: transparent colonies with black center due to H_2S production; *Shigella* spp.: without black center
**Thiosulfate-Citrate-Bile-Sucrose (TCBS) agar**	S/D	Inhibits: Gram-positive and most Gram-negative bacteria Enriches: *Vibrio* spp.	Sodium thiosulfate, sodium citrate, NaCl for Gram-negative bacteria (IA); bile salts, NaCl for Gram-positive bacteria (IA); sucrose, thymol-blue - yellow in acidic, blue/green in alkaline, bromothymol blue (I)	Sucrose fermenters (*Vibrio cholerae*): yellow colonies Non-sucrose fermenters utilize citrate (*Vibrio parahaemolyticus*): blue-green colonies
**Lowenstein Jensen (LJ) Medium**	S	Inhibits: non-mycobacterial contaminants; enriches: *Mycobacterium* spp.	Coagulated eggs, glycerol, potato flour, salts. LJ variants: Standard—malachite green (IA); Gruft—malachite green + penicillin/nalidixic acid (IA); Petran & Vera—malachite green + cycloheximide, lincomycin, nalidixic acid (IA); Stonebrinks—pyruvate replaces glycerol to boost *Mycobacterium bovis* growth.	Inoculate two media sets: 25–30 °C for *Mycobacterium marinum, Mycobacterium chelonae, Mycobacterium ulcerans*; 35–37 °C for *Mycobacterium tuberculosis* and others.<br>Colony morphology:<br>- *M. tuberculosis:* cauliflower-like, cream-colored, dry, rough, eugonic, slow-growing (2 to 6 weeks)<br>- *M. bovis:* flat, smooth, small, non-pigmented, slow-growing<br>- *Mycobacterium avium:* smooth, soft, non-pigmented, slow-growing<br>- *Mycobacterium kansasii:* smooth to rough, yellow/orange after light exposure, slow-growing<br>- *Mycobacterium gordonae:* smooth, yellow/orange regardless of light, slow-growing<br>- *M. marinum:* smooth to intermediate, white/beige in dark turns yellow/orange in light, slow-growing<br>- *M. ulcerans:* smooth or rough, clear, white or pale yellow, extremely slow-growing (4–8+ weeks)<br>- *Mycobacterium fortuitum:* smooth or rough, non-pigmented, fast-growing, <7 days<br>- *Mycobacterium abcessus:* smooth or rough, non-pigmented, fast-growing, <7 days<br>- *M. chelonae:* smooth, round, pale-cream, non-pigmented, fast-growing, <7 days
**Cetrimide agar**	S/D	Inhibits: non-pseudomonad bacteria; enriches: *Pseudomonas aeruginosa*	Cetrimide (IA); $MgCl_2$ and K_2SO_4 enhance pigment production (DA)	Pyocyanin/pyoverdine (*P. aeruginosa*): bluish-green or yellow-green colonies, metallic sheen, grape-like odor; fluoresce under 254 nm UV

**Triple sugar iron (TSI)**	D	Differentiates Gram-negative enteric bacteria based on carbohydrate fermentation (glucose, lactose, sucrose) and hydrogen sulfide (H_2S) production.	- **Carbohydrates**: glucose (0.1%), lactose (1%), sucrose (1%) (DA)<br>- **peptones** (nutrient source, IA)<br>- **Ferrous sulfate** (I for H_2S)<br>- **Phenol red** (I)<br>- **Sodium thiosulfate** (I for H_2S)	Alkaline/acid (red slant/yellow butt): glucose fermentation only (e.g., *Salmonella* Typhi)<br>- Acid/acid (yellow slant/yellow butt): glucose and lactose and/or sucrose fermentation (e.g., *E. coli*)<br>- Alkaline/alkaline (red slant/red butt): no fermentation (e.g., *P. aeruginosa*)<br>- H_2S production: black precipitate in butt (e.g., *Salmonella* spp.)<br>- Gas production: cracks/lifting of agar (e.g., *E. coli*)
**CHROMID® CPS® ELITE (bioMérieux, France)**	S/D	Isolation, differentiation, and presumptive identification of common uropathogens from urine samples; Inhibits: competing bacteria	Chromogenic substrates (DA) - enzyme-specific reactions, peptones, selective agents (IA for Gram-positive bacteria and some non-target organisms)	- ß-Glucuronidase (*E. coli*): red–burgundy colonies (direct identification)<br>- ß-Glucosidase (KESC-*Klebsiella*, *Enterobacter*, *Serratia*, *Citrobacter*): green–blue-green colonies (presumptive identification)<br>- ß-Glucosidase (*Enterococcus*): turquoise/blue colonies (presumptive identification)<br>- Indole (*Proteeae*): light–dark brown colonies (presumptive identification)
**ChromID® CARBA SMART (bioMérieux, France); CHROMagar™ mSuperCARBA (MAST Group, UK)**	S/D	Isolation and differentiation of carbapenemase -producing *Enterobacterales*	Antibiotics (IA), chromogenic substrates (DA) - enzyme-specific color reactions, nutrients	Carbapenemase producers grow with characteristic colony colors (e.g., *E. coli* – pink/red; *Klebsiella*/*Enterobacter* – blue/green); non-CPE inhibited or show reduced growth
**ChromID® ESBL (bioMérieux, France)**	S/D	Detection of ESBL-producing *Enterobacterales*	Cephalosporins (IA), chromogenic substrates (DA), peptones	ESBL producers grow with species-specific colors (e.g., *E. coli* – pink to burgundy; *Klebsiella*/*Enterobacter* – metallic blue); non-ESBL organisms inhibited
**Mueller Hinton**	N	General-purpose medium mainly for antimicrobial susceptibility testing	Beef extract, casein hydrolysate, starch, agar	Continuous surface growth; inhibition zone size around antibiotic discs indicates susceptibility

N: nonselective; S: selective; E: enriched; D: differential; IA: inhibitory agent; DA: differentiating agent; I: indicator
