# Supplementary material for: Evolving Approaches to Bacterial Identification: A Review of Classical and Modern Techniques
Source: Int J Mol Sci. 2026 Jun 4;27(11):5092. doi: 10.3390/ijms27115092 (PMC13256776; doi:10.3390/ijms27115092)
Supplement: Supplementary file 1 [file ijms-27-05092-s001.zip › Supplementary Table S2.pdf]

Supplementary Table S2. Commonly used bacterial liquid culture media and their properties [12]

Media name	Type	Purpose	Key components	Interpretation (examples)
**Nutrient broth**	N	General cultivation	Peptone, beef extract	Growth of non-fastidious bacteria
**Tryptic Soy Broth (TSB)**	N	General cultivation	Enzymatic digest of casein (tryptone), soy peptone, NaCl, sometimes dextrose	Supports growth of non-fastidious bacteria; used for inoculum preparation for automated systems and susceptibility testing; 5–10% blood may enhance growth
**Brain Heart Infusion (BHI) broth**	E	Growth of delicate, fastidious bacteria, cultivation of clinical isolates	Infusion of calf brain and beef heart, peptones, dextrose, salts	*Haemophilus* spp., *Neisseria* spp. *Listeria* spp., *Streptococcus* spp.
**Selenite broth**	S	Inhibits: Gram-positive bacteria and competing gastrointestinal flora; enriches: *Salmonella* spp.	Peptones, salts, lactose, sodium phosphate, sodium selenite (IA)	Growth indicates possible *Salmonella* spp.; turbidity shows pathogen enrichment; subculture on selective agar (e.g., XLD or SS) is needed for confirmation
**GN (Gram-negative) broth**	S	Inhibits: Gram-positive bacteria	Peptones, bile salts, antibiotics (IA)	Gram-negative bacteria
**Phenylethyl Alcohol (PEA) broth**	S	Inhibits: Gram-negative bacteria; enriches: Gram-positive bacteria	Peptones, NaCl, phenylethyl alcohol (IA)	*Staphylococcus*, *Streptococcus, Enterococcus* (often used to enrich Gram-positive bacteria before plating)
**Thioglycolate broth**	N/E	Supports growth of a wide range of bacteria and determines oxygen requirements, including aerobes and anaerobes	Sodium thioglycolate, L-cystine (reducing agents), peptone, glucose, yeast extract, NaCl, small amount of agar, resazurin (DA)	Blue/purple = oxidized (aerobic/no growth); colorless = reduced (anaerobic growth). Growth patterns: obligate aerobes—top, obligate anaerobes—bottom, facultative anaerobes—throughout (denser at top), microaerophiles—just below surface, aerotolerant anaerobes—evenly distributed
**Schaedler broth**	S/E	Enriches: strict anaerobes	Peptones, yeast extract, glucose and starch, L-cysteine, hemin, vitamin K_1	Fastidious anaerobes (often used as enrichment broth before plating on solid anaerobic media)
**Columbia broth with antibiotics**	S	Inhibits: Gram-negative bacteria; enriches: Gram-positive bacteria	Brain-heart infusion base, colistin, nalidixic acid (IA)	Gram-positive bacteria

**Blood culture media for anaerobes and aerobes: BD BACTEC™ (Becton Dickinson, USA); BacT/ALERT (bioMérieux, France);** VersaTREK **(Thermo Fisher Scientific, Cleveland, USA)**	S/E	Manual or automated anaerobic blood culture	Peptones, sodium polyanethol sulfonate (anticoagulant), and resins or charcoal to neutralize antibiotics;<br>Anaerobic bottles include reducing agents (IA) (e.g., cysteine, thioglycolate) and redox indicators (I) (e.g., resazurin).<br>Aerobic bottles are designed with oxygen-permeable headspace	Growth detected by CO_2 production, fluorescence, pressure change, or optical turbidity in automated system, or by turbidity or colony growth in manual systems
**Middlebrook 7H9 broth**	E	Enriches: *Mycobacterium* spp.	Bovine albumin, dextrose, glycerol, catalase, NaCl, KH_2PO_4, $MgSO_4$, $(NH_4)_2SO_4$, biotin, pyridoxine, $ZnSO_4$, $CuSO_4$, etc.	Turbidity indicates mycobacterial growth (can be used in MGIT system as a base for automated detection)
**BD BACTEC™ MGIT™ - Mycobacterial growth indicator tubes (Becton Dickinson, USA)**	S/E	Inhibits: faster-growing bacteria and fungi; enriches: *Mycobacterium* spp.	Middlebrook 7H9 broth with OADC (oleic acid, albumin, dextrose, catalase), PANTA antibiotics (polymyxin B, amphotericin B, nalidixic acid, trimethoprim, azlocillin) (IA), and a fluorescent oxygen-quenching sensor at the tube bottom (I)	Used in BD BACTEC™ MGIT™ automated culture system for the rapid detection of *Mycobacterium* spp.; specimens require decontamination before inoculation; bacterial O_2 consumption triggers sensor fluorescence detected by the instrument; time to positivity: 7–14 days for fast-growing mycobacteria and 10–21 days for *Mycobacterium tuberculosis*; also used for susceptibility testing

N: nonselective; S: selective; E: enriched; D: differential; IA: inhibitory agent; DA: differentiating agent; I: indicator
