# Supplementary material for: Evolving Approaches to Bacterial Identification: A Review of Classical and Modern Techniques
Source: Int J Mol Sci. 2026 Jun 4;27(11):5092. doi: 10.3390/ijms27115092 (PMC13256776; doi:10.3390/ijms27115092)
Supplement: Supplementary file 1 [file ijms-27-05092-s001.zip › Supplementary Table S3.pdf]

Supplementary Table S3. Molecular methods for bacterial detection, and species/genotype-level identification [51-88]

Category	Method / Generation	Principle	Quantification	Multiplexing	Sensitivity	Turnaround time	Cost	Key advantages	Limitations	Examples of applications
Amplification (NAATs)	**1st gen: conventional PCR**	End-point amplification + gel electrophoresis	✗	Low	Moderate/ high	3–6 h	**Low**	Simple, specific	No quantification, contamination risk, prior target knowledge required	*Salmonella* (*invA*), *S. aureus* (*nuc*), AMR (*mecA*, *bla* variants)
	**Multiplex PCR**	Multi-target amplification	✗	High	Moderate /high	3–5 h	**Low**/medium	Simultaneous detection	Primer competition	Syndromic panels
	**2nd gen:** qualitative PCR and qPCR	Real-time fluorescence detection	✗ (qualitative); semi (q PCR)	Moderate	**High**	**1–3 h**	Medium	Sensitive, closed system (low risk of contamination)	Instrument cost	*M. tuberculosis* complex (*IS6110*, *rpoB*)
	**2nd gen: multiplex qPCR**	Multi-fluorophore detection	✓	**High**	**High**	**1–3 h**	Medium /**high**	Efficient	Complex optimization	Respiratory/sepsis panels
	**3rd gen: dPCR**	Partitioned endpoint quantification	✓ Absolute	Low	**Very high**	3–6 h	**High**	Inhibitor resistant	Complex workflow	*S. aureus (nuc, mecA)*
	**3rd gen: ddPCR**	Droplet partitioning	✓ Absolute	Low	**Extremely high**	4–6 h	**High**	Inhibitor resistant	Complex workflow	Sepsis, central nervous system infections, AMR, environmental pathogens
	**3rd gen: LAMP**	Isothermal amplification	Semi	Low	**High**	**<1 h**	**Low**	Simple, point-of-care	Risk of non-specific amplification, optimisation	Field diagnostics
	**3rd gen: Lab-on-chip PCR**	Integrated microfluidic PCR	✓	**High**	**High**	**<1–2 h**	**High**	Automated	Emerging technology	Point-of-care testing
Hybridization	**DNA–DNA hybridization**	DNA complementarity	✗	Low	Moderate	**Days**	**High**	Historical gold standard	Labor-intensive	Taxonomy
	**Probe assays**	rRNA probe binding	✗	Low	**High**	**1–2 h**	Medium	Detection from samples	Limited targets	*Mycobacterium* spp.

	**Line-probe assays**	Reverse hybridization	✘	Moderate	**High**	3–5 h	Medium	Mutation detection	Target-dependent	AMR (*H. pylori*, resistance mutations)
	**Luminex/ Verigene**	Bead-based multiplex hybridization	✘	**High**	**High**	**1–2 h**	**High**	High throughput, from specimens	Complex workflow, limited panels	Blood culture ID + AMR
	**Hybridization + NGS**	Target enrichment	✘	**High**	**Very high**	Variable	**High**	Improves sequencing specificity	Prior knowledge required	Genomics, resistance profiling
**Typing**	**Ribotyping (RFLP)**	Restriction + rRNA probe hybridization	✘	Low	Moderate	**1–2 days**	Medium	Reproducible fingerprinting	Low resolution	*L. monocytogenes*, *S. enterica*, *E. coli* surveillance
	**PCR ribotyping**	Spacer region analysis	✘	Low	Moderate	**1 day**	Medium	Standardized; improved with capillary electrophoresis (enhances discriminatory power, reproducibility, ease of interpretation)	Limited discrimination	*C. difficile* typing
**Sequencing (1st gen)**	**Sanger sequencing**	Chain termination	✔	✘	**Very high**	**1 day**	Medium	Highly accurate	Low throughput	*sodA (Streptococcus), rpoB (M. tuberculosis)*
	**16S rRNA sequencing** (classically: Sanger-based; NGS-based)	Conserved + variable regions	Semi	**High**	**High**	**1–2 days**	Medium	Broad ID	Limited species resolution	Unknown bacteria
**Sequencing (2nd gen – NGS)**	**Short-read sequencing**	Massive parallel sequencing (≤600 bp)	✔	**Very high**	**Very high**	**1–3 days**	Medium /**high**	High throughput	Short reads	Genomics
	**WGS (NGS)**	Whole-genome sequencing	✔	**High**	**Very high**	**2–5 days**	**High**	Full genomic characterization	Bioinformatics complexity	Outbreaks, AMR, virulence
	**SNP analysis**	Genome comparison	✔	✘	**Very high**	Variable	Medium	High resolution typing	Requires reference genome	Clonal tracking
	**cgMLST / wgMLST**	Allelic profiling	✔	✘	**Very high**	Variable	Medium	Standardized typing	Computationally demanding	Surveillance

	**Amplicon sequencing**	Targeted sequencing	Semi	**High**	**High**	**1–2 days**	Medium	Efficient profiling	Limited scope	Microbiome studies
	**Shotgun metagenomics**	Total DNA sequencing	✓	**Very high**	**High**	**2–5 days**	**High**	Species + genes	Broad detection (species + genes), complex	AMR, microbiome
**Sequencing (3rd gen – TGS)**	**Long-read sequencing**	Single-molecule sequencing (>1–100 kb)	✓	Moderate	**High**	**1–2 days** (or real-time)	**High**	Resolves repeats, plasmids	Higher error rate	Genome assembly
	**Real-time sequencing**	Continuous reading	✓	Moderate	**High**	Hours	**High**	Rapid results	Data complexity	Clinical genomics
	**Portable sequencing**	Field-based sequencing devices	✓	Moderate	Moderate/ **high**	Hours	Medium /**high**	On-site testing	Variable accuracy	Public health
	**Hybrid (NGS + TGS)**	Combined sequencing reads	✓	**High**	**Very high**	**2–5 days**	**High**	Highest accuracy	Complex workflow	Outbreak investigation

Gen: generation; PCR: polymerase chain reaction; qPCR: quantitative PCR; dPCR: digital PCR, ddPCR: droplet digital PCR; NGS: next generation sequencing; AMR: antimicrobial resistance; TGS: third generation sequencing; Green indicates key advantages; red indicates major limitations.
