# Supplementary material for: Evolving Approaches to Bacterial Identification: A Review of Classical and Modern Techniques
Source: Int J Mol Sci. 2026 Jun 4;27(11):5092. doi: 10.3390/ijms27115092 (PMC13256776; doi:10.3390/ijms27115092)
Supplement: Supplementary file 1 [file ijms-27-05092-s001.zip › Supplementary Table S4.pdf]

Supplementary Table S4. Core quality control strains routinely used in microbiological veterinary laboratory

Bacterial species	ATCC No.	Primary quality control purpose	References
CORE QC STRAINS			
Escherichia coli	25922	AST; enteric isolates	[109]
Staphylococcus aureus	25923	Mastitis; disk diffusion	[109]
Staphylococcus aureus	29213	MIC	[109]
Pseudomonas aeruginosa	27853	Non-fermenter	[109]
Enterococcus faecalis	29212	AST; enteric isolates	[109]
BOVINE MASTITIS QC STRAINS			
Staphylococcus aureus	25923	Coagulase +; mastitis	[109]
Streptococcus agalactiae	13813	Contagious mastitis	[110]
Streptococcus uberis	19436	Environmental mastitis	[110]
Escherichia coli	25922	Coliform mastitis	[109]
SWINE & LIVESTOCK RESPIRATORY PATHOGENS			
Pasteurella multocida	12945	Respiratory pathogen	[110,111]
Mannheimia haemolytica	33396	Bovine respiratory	[112]
Actinobacillus pleuropneumoniae	27090	Swine pleuropneumonia	[110,111]
POULTRY PATHOGENS			
Salmonella enterica ser. Typhimurium	14028	Selective media & biochemical	[113]
Salmonella enterica ser. Enteritidis	13076	Poultry	[110,114]
Escherichia coli	25922	Avian colibacillosis	[109]
COMPANION ANIMAL PATHOGENS			
Staphylococcus pseudintermedius	49444	Canine pyoderma	[109]
Proteus mirabilis	12453	Canine urinary tract infections	[109]
Klebsiella pneumoniae	700603	ESBL production	[109]
ANAEROBIC & SPECIAL PATHOGENS			
Clostridium perfringens	13124	Enterotoxemia	[110,115]
Bacteroides fragilis	25285	Anaerobic	[108,109]

Brucella abortus	23448	Serology & identification	[116]
**ANTIMICROBIAL RESISTANCE**			
Escherichia coli	35218	β-lactamase production	[109]
Staphylococcus aureus	43300	MRSA	[109]
Enterococcus faecalis	51299	VRE	[109]

AST: Antimicrobial susceptibility testing; ESBL: Extended-spectrum beta-lactamases; MRSA: Methicillin-resistant *Staphylococcus aureus;* VRE: vancomycin-resistant *Enterococci*
